# Belonging and Social Integration as Factors of Well-Being in Latin America and Latin Europe Organizations

**DOI:** 10.3389/fpsyg.2020.604412

**Published:** 2020-12-09

**Authors:** Silvia da Costa, Edurne Martínez-Moreno, Virginia Díaz, Daniel Hermosilla, Alberto Amutio, Sonia Padoan, Doris Méndez, Gabriela Etchebehere, Alejandro Torres, Saioa Telletxea, Silvia García-Mazzieri

**Affiliations:** ^1^Department of Social Psychology, Faculty of Psychology, University of the Basque Country, San Sebastian, Spain; ^2^Departament of Social Psychology, Faculty of Labour Relations and Social Work, University of the Basque Country, Leioa, Spain; ^3^Departament of Social Psychology, Faculty of Labour Relations and Social Work, University of the Basque Country, Vitoria, Spain; ^4^Departament of Psychology, Faculty of Psychology, University of Talca, Talca, Chile; ^5^Institute of Psychology, Education and Human Development, Faculty of Psychology, University of the Oriental Republic of Uruguay, Montevideo, Uruguay; ^6^Argentine National Defense University, Buenos Aires, Argentina; ^7^Departament of Psychology, Regional Faculty of the National Technological University, Trenque Lauquen, Argentina

**Keywords:** belonging, factors, organizations, social integration, well-being

## Abstract

**Background:**

Studies and meta-analyses found individual, meso and micro-social factors that are associated with individual well-being, as well as a positive socio-emotional climate or collective well-being.

**Aim:**

This article simultaneously studies and examines these factors of well-being.

**Method:**

Well-Being is measured as a dependent variable at the individual and collective level, as well as the predictors, in three cross-sectional and one longitudinal studies. Education and social intervention workers (*N* = 1300, *K* = 80) from Chile, Spain and Uruguay participate; a subsample of educators (*k* = 1, *n* = 37) from the south central Chile and from Chile, Uruguay and Spain (*n* = 1149); workers from organizations in Latin America and Southern Europe, military cadets from Argentina (*N* < 1000); and teams (*K* = 14) from Spanish companies.

**Results:**

Individual and collective well-being indicators were related, suggesting that the emotional climate as a context improves personal well-being. Individual factors (emotional creativity and openness and universalism values), psychosocial factors (low stress, control over work and social support supervisors and peers) were positively associated with personal well-being in education and social intervention context. Organizational dynamic or transformational culture is directly and indirectly associated with individual well-being through previously described psychosocial factors. Group processes such as internal communication and safe participation, task orientation or climate of excellence as well as leadership style that reinforces participation and belonging, were positively associated with collective well-being in labor and military context and predict team work socio-emotional climate in a longitudinal study- but were unrelated to individual well-being. Transformational leadership plays a mediating role between functional factors and social-emotional climate in work teams. Organizational role autonomy, functional organizational leadership, integration and resources were associated with collective well-being in organizations. Organizational leadership moderates the relationship between task orientation and collective well-being in military context.

**Conclusion:**

Individual and microsocial factors influence personal well-being. Meso level factors favorable to well-being through processes which reinforce social belonging, influence directly collective well-being and indirectly personal well-being. Leadership that reinforces participation and belonging play a central role for emotional climate. Stress and emotional climate playing an important pivotal role for psychological well-being.

## Introduction

This article examines the relative importance of factors in well-being in Latin America and Southern European organizations. We briefly review the theoretical antecedents of well-being in organizations, which will then be examined empirically in the following four studies in students and workers from six countries. The International Labor Organization (ILO)^1^ states that well-being at the workplace concerns all aspects of professional life. In this sense, the quality and safety of the physical climate, the socio-emotional climate and work organization are of great importance. There are studies that report a direct relationship between productivity levels, health and the general well-being of the workforce (Martín, 2011; Steffens et al., 2016; Kickbusch et al., 2017; World Health Organization, 2020). Regarding the well-being of organizations, this implies processes such as collective identification and social integration, as well as multi-level efforts. The concept of identification with the organization implies a perception of unity or belonging to it. As such, this would be a determining variable to explain desirable consequences therein (Mota et al., 2018). When belonging to an organization is part of an individual’s social identity, its norms and values are incorporated into the cognitive concept of the self, as well as the attraction and/or desire to belong to it (Davila and Jiménez, 2012). In turn, we understand that social integration (Hirsch et al., 2008) is a multidimensional construct that can be defined as the extent to which individuals participate in a variety of social relationships, like labor roles and organizations (Holt-Lunstad et al., 2010), and this includes cohesion, group identification (Knight and Eisenkraft, 2015), support and social capital (Hirsch et al., 2008). According to the theory inspired by Durkheim (Hirsch et al., 2008; Song, 2013), well-being would be in proportion to the degree of social integration of individuals in the groups of which they form a part (Song, 2013). Thus, social integration is associated with a lower risk of mortality (Holt-Lunstad et al., 2010, 2015) and greater health behaviors (DiMatteo, 2004; Holt-Lunstad et al., 2010, 2015).

### Criteria for Membership and Social Integration in Organizations

There are multiple factors at different levels that explain well-being in organizations^2^. As an individual-level dependent variable (DV), this article reviews well-being indicators like stress reactions (BSCs), the concept of quality of life linked to health (QLLH), the hedonic view or subjective well-being (SHWB), psychological or eudemonic well-being (PWB), and social well-being (SWB). In turn, as micro-social level DV, we study the concept of the socio-emotional climate, regarding hedonic group well-being, related to the predominance of positive collective emotions and social cohesion. As individual-level explanatory variables, we review emotional creativity (EC) and motivational values (MV) (Schwartz, 2012), such as openness to change (OVC) and self-transcendence benevolence (TVB) and universalism (TVU). In the same fashion, we examine gender, seniority with the organization (SO), degree of knowledge and previous participation in work teams (KPW), agreement with the methodology (AM) used at the organization and the intention to stay (IS) at it. The latter two are, respectively, indicators of professional satisfaction and commitment to the organization. As explanatory variables of micro-social level, we examine group processes that facilitate integration and participation. That is, the internal participation and communication within the group (IPaCG), and task-orientation and a climate of excellence (TOaCE). At this level, we also analyze the concepts of excessive psychological demands at work or job-place stress (EPs), control over work or autonomy in one’s position (CWa) and leadership that reinforces participation and belonging to the organization (LpB) which includes the different leadership styles. Finally, and as variables of meso social level, we examine the characteristics of the labor role (AR and LeR), the culture and organizational structure that reinforces participation and integration in the organization (CSO). This includes transformational and transactional culture and organizational leadership (LpO).

### Well-Being as an Explained Variable at the Individual and Micro-Social Level

At an individual level, two basic elements of well-being are BSCs and QLLH. BSCs are different forms of behavior that are sometimes related to stress. Symptoms such as dry mouth, tendency to perspire, stomach pain, and more, may be explained by the physiological alterations that occur in the body when the “fight-or-flight” response is activated. Long-term stress has a negative effect on the health of the individual who suffers it. It may make itself known only through experiential symptoms of an emotional variety (nervousness, irritability, distress, anger, etc.) or through the cognitive and behavioral consequences related to it, including greater risk of traffic accidents, lethal decisions and erroneous decisions at work. On the other hand, QLLH is defined as the score an individual gives to his/her degree of well-being in different areas of life, considering the impact that a disease and its consequences may have on the aforementioned areas. This includes perception of physical and mental health and vitality. Thus, positive mental health implies a lack of symptoms of anxiety and depression. Good mental health in professional terms is related to quality of leadership, predictability, social support and meaning of work. In this regard, vitality is very similar to “joie de vivre” and has been shown to have a high negative correlation with feeling burnout on the job (Hervás and Vázquez, 2013; Moncada et al., 2014).

The SHWB or subjective well-being falls under a hedonic vision of well-being. We can identify both the affective and the cognitive aspects of this focus (Sojo et al., 2016; Martín-María et al., 2017; Diener et al., 2018). AHWB refers to a person’s experience of pleasant and unpleasant feelings (Tov, 2018). SWL has often been called life satisfaction, which is a judgmental process, wherein the person assesses quality of life based on his/her own criteria (Vanhoutte and Nazroo, 2016). EWB or eudaimonic well-being includes acceptance and appreciation of oneself, or self-esteem, having positive relationships with other people, feeling capable of effectively working and acting, and that one is learning or undergoing personal growth, that includes a purpose in life and a feeling of personal psychological and social autonomy (Suh and Koo, 2008; van Tuin et al., 2020). For its part, social well-being (SWB) refers to the extent to which one’s surroundings provide for a full life or facilitate realization of the most valuable human potential (Ryan et al., 2008; Mackenzie et al., 2018). Finally, EPWB, or psychological well-being is composed by SHWB, EWB and SWB (Ryan and Deci, 2001; Hervás and Vázquez, 2013; Ryff, 2014).

At microsocial level, well-being could be conceived of as group hedonic well-being (GHWB). Organizational climate has been defined as the relatively shared perception of interactions and their meanings that characterize a group or organization. Organizational psychology defines socio-emotional climate as “a particular form of organizational climate that specifically refers to the collective mood of the members of the organization and their attitudes toward their colleagues and leaders, as well as the organization as a whole. In this regard, the climate, although related to the culture of the organization, is different from it, since it is a function of the political organization and organizational procedures, as opposed to the beliefs, values and suppositions of its members” (Ashkanasy and Dorris, 2017, p. 79). The emotional culture of the group, organization or nation are the shared affective values, norms, mechanisms, scenarios and suppositions that govern the emotions that people must feel and express (Páez et al., 2013; Barsade and Knight, 2015; Bobowik et al., 2017). The positive climate is characterized by positive emotions, which are created and then fed by the organization’s structure (Ashkanasy and Dorris, 2017). Three aspects of teamwork, closely related to GHWB, are trust, bonding and satisfaction with participation. The first two refer to the establishment of bonds of affection on the team. Trust assesses the extent to which members have confidence in the team’s ability to carry out their task and help each other. On the one hand, it is based on the team’s power, or the collective belief that they will be able to successfully complete the task (Tröster et al., 2014). On the other hand, it is based on the psychological certainty or belief, held by each one of the members, that they will not be threatened and/or rejected from the team (Hülsherger et al., 2009; DeChurch and Mesmer-Magnus, 2010). At the same time, bonding is based on managing diversity as a source of resources, and conflict from a cooperative perspective, in search of mutual benefit. By last satisfaction with participation refers to how much team members like their colleagues and the team in general (Johnson and Avolio, 2019; Settles et al., 2019). A meta-analysis found that positive group affect^3^ was consistently associated with social integration and task performance (Knight and Eisenkraft, 2015).

In line with the aforementioned, the following hypotheses are proposed:

**H1:** Confirming the nomological well-being network, the indicators of individual level and collective level will be associated with each other. Demonstrating the independence of the constructs, the associations between well-being indicators will be less than 0.70.

### Predictors of Well-Being at the Individual, Micro and Mesosocial Level

EC is understood as the ability to experience novel and complex combination of emotions in an appropriate, authentic and original way (Averill, 2009). Emotional intelligence is a similar concept (Gong and Jiao, 2019). While EC requires divergent thinking, where the process and generation of an adequate response are just as important as originality, EI requires convergent thinking and solving emotional problems so the experience is recognized with precision (O’Connor et al., 2019). EC has been found to be positively related to positive emotional experiences, like positive affect and hope (Sharma and Mathur, 2016), as well as self-esteem (Oriol et al., 2016). In the same way EC was also associated with a high intensity of negative affect, which is congruent with the fact that EC is associated with neuroticism, a personality trait linked to emotional reactivity and negative affect (Averill, 1999; Runco, 2014). However, EC, which is associated with a more adaptive hetero regulation (da Costa, 2018), very likely is also associated with a more positive socio-emotional climate, helping to decrease the negative emotions of others and increase the positive ones. MVs (Castro et al., 2017) are guides to the actions of people and their personal interests. For this reason, they do not always align with the organizations. These values may be viewed as orientations for action and meet one or more needs: biological, for coordinated social interaction, for survival and for well-being of groups (Schwartz, 2012; Castro et al., 2017). OVCs (which emphasize self-direction, hedonism, and stimulation), linked to needs of self-determination and competency, as well as orientation toward variety and gratification, have been associated with the well-being of individuals. Thus TVUs, oriented toward justice and well-being for all individuals, as well as TVB, which emphasizes social support for those near to us, have been associated with well-being (Bobowik et al., 2011; Zubieta et al., 2012; Schwartz and Sortheix, 2018).

Regarding gender, the meta-analysis by Batz-Barbarich et al. (2018) found no statistically significant differences in satisfaction with life and for satisfaction with work between genders Once the publication bias was corrected, differences were significant (*d* = −0.03 for life satisfaction and *d* = −0.011 for job satisfaction) but small, in favor of men. The meta-regression had an effect size of *r* = −0.12 for gender inequality in female well-being. Another meta-analysis (Purvanova and Muros, 2010), which studied the relation between gender and burnout, found women slightly more emotionally burned out than men (δ = 0.10), and men were slightly more de-personalized than women (δ = −0.19). According to Batz and Tay (2018), most of the meta-analysis’ results conclude that gender differences are significant in terms of satisfaction with life, with men having higher life satisfaction levels. A study (*K* = 154 countries) suggested that women report higher levels of negative affect than men. One possible explanation is that an underlying related theme is the responsibility of caring for dependents, since women often assume the role of primary caregivers (Sharma et al., 2016; Chawla and Sharma, 2019).

Regarding SO, the results from a survey conducted with employees in the United States and Korea showed that the positive relationship between people-oriented leadership and affective organizational commitment were moderated by rank and years at the organization. It was observed that the positive relationship was stronger when the rank was higher and the SO was shorter (Hong et al., 2016). Peiró et al. (2019) found that, at Spanish companies, the likelihood of obtaining high well-being and performance was associated with a temporary contract (see De Neve and Ward, 2017), being between 35 and 50 years old and playing a managerial role (see intention to stay). Finally, Romero (2001) found that younger workers with fewer years at the organization showed greater psychological well-being and job satisfaction, while other studies found the opposite (Oshagbemi, 2000) or a U shaped association (Ronen, 1978). As for KPW, a longitudinal study (Nielsen and Randall, 2012) found that levels of autonomy and satisfaction in the job, before the intervention, predicted the degree of employee participation in planning and executing the intervention. Participation and changes in work procedures were significantly associated with well-being after the intervention. Another study found that knowledge about teamwork had an impact on the team’s results, and that team learning behavior mediated between knowledge of teamwork and its results (Guchait et al., 2015). AM was conceived as a job-satisfaction indicator that could be defined as the individual’s psychological willingness toward their job which involves an emotional or affective response (Cantarelli et al., 2016). The meta-analysis by Bowling et al. (2010) found at the same time a positive relationship between job satisfaction and life satisfaction, subjective well-being and positive affect. Finally, IS can be defined as the desire of those working at an organization to stay there (Ogbonnaya et al., 2018) and in the professional sector (da Costa, 2018), with a high degree of relevant implication in organizational effectiveness. Ratifying this, a multi-level analysis found an indirect positive association in patients’ satisfaction through well-being of employees and intention to stay. The strength of this relation appears to be reinforced by the training that the organization provided to its employees (Ogbonnaya et al., 2018).

The following hypotheses are formulated:

**H2:** Emotional creativity, Openness to change and Self-transcendence values will be positively associated with well-being.

**H3**: According to the theory, gender differences in well-being will be found to explain less than 3% of the variance (*d* < 0.20 or *r* < 0.10) and those that are found will benefit men. Following the theory, we also postulate that differences found in pro-well-being factors will sometimes favor men and other women.

**H4:** The less seniority and the greater knowledge about team work, commitment and job satisfaction the greater the well-being.

At a microsocial level, IPaCG is a process related to belonging and social integration. This is characterized by participation in decision-making and communication between group members and with the organization. When people can participate in decision-making, they have influence and feel free to speak, they display greater commitment and they tend to invest more energy in their work. In this regard, open and fluid communication encourages a non-threatening psychological climate, characterized by comradeship and mutual support. Another communication method is through ICTs. Work teams use them to share information quickly and effectively amongst members. To achieve high coordination for better performance and results, it is important that members have the same degree of knowledge and mastery over them (Müller and Antoni, 2020). TOaCE refers to a climate of excellence, describing the shared concern for excellent quality in conducting tasks, associated with a shared vision or results. Task orientation includes the process wherein the team reflects on its objectives, strategies, procedures and processes and assesses each individual’s work to improve efficacy and coordination (Hülsherger et al., 2009). Team member coordination may be explicit [visible and external coordination patterns (Chang et al., 2017), or implicit (Rico et al., 2008)], referring to team members’ knowledge, their experience in conducting a certain task, and how to efficiently integrate this knowledge (Butchibabu et al., 2016; Bachrach et al., 2019). It has been proven that team coordination is a group process that increases team performance over time (Marques-Quinteiro et al., 2019; Rico et al., 2019; Braun et al., 2020)^4^. The review by Nielsen et al. (2017) found that the correlation of group-level variables with well-being was significant, *r* = 0.25.

The concept of CWa, implies influence (or autonomy) and skill development (Chiang et al., 2013). Autonomy means that members participate in deciding on the work methods used by management, the possibilities for development, the opportunities afforded by conducting a task to put members’ skills into practice and the possibility of acquiring new skills. On the one hand, this has to do with the levels of complexity and variety of tasks. On the other hand, the work has meaning if it helps to positively tackle their demands (Moncada et al., 2014). Autonomy as an organizational resource (*K* = 54), correlated (*r* = 0.31) with well-being (Nielsen et al., 2017). Adaptation is a behavioral emergent (DeChurch and Mesmer-Magnus, 2010) referring to autonomy and control over work at a team level. In general, the team uses cognitive, verbal and behavioral activities to organize task activities. In turn, the team can assess the situation of adaptation, learn what it needs to meet demands and draw up strategies and responses to improve adaptation or to make it more satisfactory. The team’s adaptive performance emerges as the members conduct the different tasks and display different types and amounts of actions while carrying out those tasks (Christian et al., 2017). Previous studies show that this capability is the best predictor of a team’s performance (DeChurch and Mesmer-Magnus, 2010; de Wit et al., 2012).

EPs are an organizational process that may hinder social integration and well-being. Its quantitative aspect refers to the work volume in relation to the time available to complete it. The qualitative aspect considers that psychological requirements are different depending on whether one works with and for people, thus defining psychological requirements as emotional (Moncada et al., 2014). In occupational hazard prevention, psychosocial factors are health hazards that originate in work organization, generating responses that can be risk factors for health. Stress, chronic tension, and events that lead to negative changes have been associated with low well-being (Schneiderman et al., 2005).

Regarding LpB, the quality of exchanges between leaders and members has been researched as a facilitator for well-being. High-quality or positive behavior of supervisors includes a willingness to listen and show support, respect, and interest in members’ well-being. It also includes a tendency to value and express support for the employees’ work. Transformational leadership leads collaborators to perform beyond their expectations, going above and beyond their own interests for the good of the organization (Avolio et al., 2009; Molero et al., 2010; Hermosilla et al., 2016). The description of this style of leadership is based on the effects that the leader causes in their followers; thus, it is considered broader and more effective than transactional leadership or a lack of leadership. Conceptually, transformational leaders set out to act as an example to be followed (charisma), giving meaning to the actions of their subordinates (inspiration), encouraging the search for alternative solutions to everyday problems (intellectual stimulation), and they tend to be concerned about the individual needs of their subordinates (Nader and Sánchez, 2010; Banks et al., 2016). Another leadership style is transactional, which uses rewards and negotiation with subordinates in exchange for reaching organizational objectives and goals. Those who use this style tend to closely supervise their subordinates’ activities to prevent possible errors or deviations from established norms and procedures (Arnold, 2017).

In most studies on organizational settings, social support (Taylor, 2011; Chawla and Sharma, 2019) was focused on the support provided within the organization. A lack of support from superiors has to do with a lack of specific staff management principles and procedures to guide this role to act as an element to support the work conducted by the team or department they manage. It is also related to a lack of clear guidelines and training regarding fulfillment of this role (Moncada et al., 2014). Meta-analyses found that supervisor social support in the workplace was associated with life satisfaction, job satisfaction and health (Kossek et al., 2011; Eby et al., 2013; Mathieu et al., 2019). It was also found that is crucial in buffering the effect of work-related stress on perceived health, and increasing the physical and mental health among military personnel (Hsieh and Tsai, 2019). The quality of leadership has to do with staff management principles and procedures, as well as training and available time for managers to apply them. The correlation of leadership-level variables (of quality studies and transformation *k* = 7) with well-being was significant, *r* = 0.27 (Nielsen et al., 2017). To underline this, another meta-analysis found that high-quality relationships between the supervisor and the employee were positively associated with well-being (*r* = 0.35) (Huell et al., 2016). Finally, emotionally expressive leaders (e.g., charismatic or transformational) induce group members to experiment and express positive emotions of high activation that are easily communicated and lead followers to experience a positive emotional climate (Barsade and Knight, 2015; da Costa, 2018).

EMPW can be recognized as a manifestation of quality leadership. As such, it refers to the extent to which the team coordinator promotes the participation of members in the team. Colleagues who feel empowered will be more effective (Paolucci et al., 2018) and will develop more proactive and innovative behaviors (Huang, 2017), improving both their own creativity and the implementation of the ideas that are generated (Rhee et al., 2017). Finally, SL is the team’s capability to distribute leadership amongst its members in opposition to of centralizing it in one sole individual (Nicolaides et al., 2014). This is viewed as the “dynamic and interactive process between the members of a team whose objective is for some to address the others to achieve the goals of the team, of the organization, or of both (Wang et al., 2011; Drescher et al., 2014; Uhl-Ben et al., 2014). At this point one might say that a characteristic of shared leadership is promotion of greater trust (Nicolaides et al., 2014) and cohesion (Mathieu et al., 2015) in the team. Moreover, it has been demonstrated that it improves satisfaction and well-being with the team (Montano et al., 2017). Studies that examined the impact of leadership on the organizational climate, found a large positive effect. This suggest that leadership style play a pivotal role dealing with organizational process and in the establishment of a positive climate (Pérez Vallejo and Fernández Muñoz, 2020).

Taken into account the aforementioned variables and ideas, the following hypotheses were proposed:

**H5**: Low psychological demands or work stress, high control over work, leadership that reinforces participation and belonging, internal participation and communication in the group, task orientation, and climate of excellence, will be positively associated with both individual and collective well-being.

**H5a:** At the team level, leadership that reinforces participation and belonging will mediate between task orientation and climate of excellence and collective well-being.

The LR provides freedom and independence to members to determine which procedures must be used to conduct the task, and how they can increase the likelihood of successful implementation of above task (Hammond et al., 2011). The meta-analysis conducted by Stewart (2006) in Hülsherger et al. (2009) found that autonomy, along with coordination within the team, contributed to better performance. Thus, another key variable that may be related to well-being are: predictability, role clarity, and professional demands. The first one predictability refers to adequate and sufficient information and that is on time, in order to be able to properly complete the job and adapt to changes. The second one role clarity implies knowledge of one’s own job position and the positions of people in the organization, the tasks to be completed, objectives, existing resources, and responsibilities beyond professional autonomy. Finally, contradictory professional demands, which may entail professional or ethical confrontations, generally unleash role conflict (Moncada et al., 2014). The expectation of the emotional labor role (LeR) refers to the fact that employees must display appropriate (positive or negative) emotions to clients or consumers. According to this, in retail, food, travel and entertainment industries, there is an expectation that employees must display positive emotional expressions or provide “service with a smile.” On the other hand, with other jobs (police, debt collector), it is expected that employees display negative emotions. A neutral or a poker face is expected with jobs related to health and treating serious diseases, or at court. Thus, there is clear evidence that emotional work, particularly hiding the emotions one feels or expressing dissonant emotions, has harmful effects on well-being and health (Ashkanasy and Dorris, 2017; Diener et al., 2020). In this sense, the meta-analysis by Hülsherger and Schewe (2011) found that superficial performance or simulating emotions that are not felt is related to a negative mood, emotional exhaustion or burnout and decreased job satisfaction.

Regarding CSO, it should be noted that while organizational culture (OC) refers to beliefs, values and ideologies that are shared by members of one same organization (Schneider et al., 2013; Hofstede et al., 2014, the OS in this case) has to do with the integration that exists at the organization, as well as available resources, to appropriately carry out work at said organization. Bass and Avolio (1992) in Nader et al. (2006) consider that organizational cultures can be characterized based on their predominant leadership style. In general, transformational cultures have a sense of purpose, constantly redefining their mission, vision, rules and principles, and their commitments are long-term ones. In these organizations, leaders and followers share interests and a sense of shared destiny and are inter-dependent. The team’s and organization’s well-being is more important for leaders and followers than their own interests and benefit. Moreover, the managers act as mentors, trainers, behavioral models, and leaders (Bass and Avolio, 2007). They are characterized by having a flexible structure and so, they tend to be more informal and dynamic, encouraging teamwork and personal growth at the same time (Belbin, 2010a, b). This type of culture has been associated with fewer role conflicts and greater organizational efficacy (Vázquez Alatorre, 2013; Ayestarán, 2016; Hartnell et al., 2019). In contrast, transactional cultures are mainly focused on terms of explicit and implicit contractual relations and tend to be very bureaucratic and structured. In these organizations, commitments tend to be short-term ones, and individual own interests prevail over the collectives. There is greater presence of role conflicts, with less organizational efficacy (Vázquez Alatorre, 2013).

Regarding organizational integration and resources, we might point out that the former is related to how different company departments coordinate their operations. A highly integrated organization has strong connections between departments and teams, and so, each section works under a coherent set of rules and strategies. Integration is associated with performance, although it can also be associated with well-being, since highly integrated organizational cultures may instill a similar feeling of social or collective identity (such as Japanese corporations, for example) (Dextras-Gauthier et al., 2012). Moreover, those organizations that tolerate uncertainty and mistakes become a less stressful context for employees. Finally, the existence of material and human resources facilitates a quality work environment (Winter et al., 2014; Hartnell et al., 2019).

Adding up to this, and as far as LpO is concerned, while direct leadership is characterized by face-to-face interactions with members of a group or team, which reinforces identification, cohesion and group learning, organizational leadership indirectly influences many more people indirectly. Thus, organizational leadership is linked to development, use of resources, organizational learning, human resources management to improve human capital (da Costa et al., 2014), reinforcement of commitment, and organizational climate (Fischer et al., 2017). This stands for leadership behaviors oriented toward reinforcing trust and a quality relationship with subordinates were associated (*r* = 0.31) with psychological well-being (Dunst et al., 2018).

From the perspective of explanation in social psychology through the articulation of levels of analysis, micro social factors are embedded in organizational context, and these psychosocial factors mediates and explain the influence of organizational culture on well-being (Doise and Valentim, 2015). Finally, Study 3 is conducted in a military institution and therefore we must take into account its organizational culture. Because military’s organizational culture is masculine and oriented towards toughness and manliness (Hofstede, 1988 in da Costa, 2018), it is expected that the functional processes within it will be more beneficial to male than female cadets.

**H6**: Low emotional labor role, high autonomy role, culture and organizational structure that reinforce participation and integration and functional organizational leadership will be positively associated with the individual and collective well-being. In addition, we postulated that the transformational style of culture and leadership will be associated more strong and positively with the well-being than the transactional.

**H6a**: Low psychological demands or work stress, high control over work and leadership that reinforces participation and belonging mediate the positive relationship between culture and organizational structure that reinforces participation and integration and individual well-being.

**H6b**: In military culture, the positive relationship between task orientation and the climate of excellence with collective well-being will be moderated by organizational leadership that reinforces participation and belonging as well as by gender (male will benefits more), enhancing the association between them. In the same vein, it is expected that gender will moderate the relationship between leadership and collective well-being.

## Materials and Methods

The studies were conducted between 2015 and 2018 in 6 Ibero-American countries: Argentina (E3, MS and WS), Brazil (E3, WS), Chile (E1, 2 and 3, WS), Spain (E1, 2, 3 and 4, WS), Mexico (E3, WS), and Uruguay (E1 and 2, WS).

### Study 1: Psychosocial Favorable Factors to Well-Being in Three Countries: Educational and Social Intervention Organizations

This study seeks to contrast the postulated associations between individual indicators of well-being: behavioral, somatic and cognitive reactions to stress (BSCs), quality of life linked to health (QLLH), affective hedonic view of subjective well-being (AHWB), cognitive hedonic view of subjective well-being or satisfaction with life (SWL), eudaimonic vision or psychological well-being and personal optimal development (EPWB) as well as to show the relative independence between them in a sample of teachers and social intervention educators from three countries (H1). It also seeks to contrast hypotheses 2–6, i.e., to test the positive association between individual well-being (EPWB) and emotional creativity (EC), values of openness to change (OVC) and transcendence values (TV) (H2), gender differences (H3) as well as positive correlation of well-being with low seniority in the organization (SO); high agreement with the methodology (AM) in the workplace and intention to stay (IS) in your organization for the long term (H4). We also want to contrast the positive relationship between psychosocial factors (EPs, CWa, LpB – social support and quality leadership) and EPWB (H5); as well as the negative relationship between EPWB and emotional labor role (LeR) and the positive association with an organizational culture that reinforces participation and integration. By doing so, it is expected that a transformational organizational culture will be associated positively and more strongly with well-being than a transactional organizational culture (H6). Finally, in this study we want to verify that the above-mentioned psychosocial factors (EPs, CWa, and LpB) mediate between a culture that reinforces participation and integration (CSO) and individual well-being (EPWB) (H6a).

#### Sample

Participants *N* = 1300 subjects (*N* = 1084 women, aged between 19 and 69 years, *M* = 41, 41, *SD* = 11, 09), who belonged to *K* = 80 organizations or educational and social-intervention units in Chile, Spain (Autonomous Community of the Basque Country or CAPV) and Uruguay. The sample of educational professionals doubled the social intervention sample^5^.

#### Procedure

The individuals received a booklet with different scales to be answered on paper or online for a period of between 50 and 75 min (only some of them are addressed in this article). Data collection was supervised by some of this article’s co-authors or staff trained to do so in each one of the three countries.

#### Instruments

The BSCs and QLLH are measured with 12-item scales, respectively (see instruments on Table 2 of Supplementary Material). The first includes behavioral, somatic, and cognitive symptoms of stress (Setterlind, 2001 in Moncada et al., 2014); the second includes dimensions of perceived physical health, mental health and vitality (Ware and Sherbourne, 1992). They both use a *Likert-like* scale with 5 anchor points (always = 1 to 5 = never) for BSCs, and for the first dimension (perceived physical health) of QLLH (totally true = 1 to 5 = totally false). Another two dimensions (mental health and vitality) are answered with 6 anchor points (always = 1 to 6 = never). Cronbach’s α:0.98 for BSCs and QLLH > 0.70 for the three dimensions. AHWB is measured with 20 items (10 positive affectivity and 10 negative affectivity) (Fredrickson, 2013), using a Likert-like scale with 5 anchor points (nothing = 0 to 4 = a lot). They were asked how they felt during the last week of their professional activity. Cronbach’s α: >0.80 for both dimensions. SWL is measured through 5 items and 10 anchor points (very unsatisfactory = 1 to 10 = very satisfactory). This instrument (Diener, 1996) is designed to assess an individual’s degree of satisfaction with certain aspects of their life, such as work, income, family, their person and life in general. Cronbach’s α:0.70, although each domain is specific to itself. Finally, EPWB is measured with the instrument by Hervás and Vázquez (2013), consisting of 11 items related to different areas of well-being, meaning hedonic, eudaimonic and social. A Likert-type scale is used for responses (totally disagree = 0 to 10 = totally agree). Cronbach’s α:0.85 for samples from the three countries.

EC is measured with a 17-item scale with 6 anchor points (totally disagree = 1 to 6 = totally agree). The instrument by Soroa et al. (2015) is adapted to Spanish from the scale by Averill’s. Cronbach’s α: 0.82. OVC and self-transcendence (Schwartz, 2012) as TVU are measured with 9 items and 6 anchor points (that sounds a lot like me = 6 to 1 = that does not sound like me at all). Cronbach’s α:0.82 (openness to the experience) and 0.87 (universalism). To measure psychosocial factors EPs, CWa, and LpB, 26 items and 5 anchor points are used (always = 4 to 0 = never). For this study, dimensions 1, 2, and 4 of CoPsoQ-Istas21 were used (Moncada et al., 2014). Cronbach’s α: 0.70 for the three dimensions. The emotional work entailed by LeR (Ortiz et al., 2012) is also measured through 21 items and 5 anchor points (very rarely = 1 to 5 = very frequently). Cronbach’s α: 0.83. Finally, the CSO (transformational and transactional culture) is measured through 28 items (Nader et al., 2006), using a dichotomous scale (Spearman Brown: <0.70, it was necessary to eliminate items 1, 7 and 19 from transactional culture in the three samples). Finally, gender, SO, AM, and IS were measured (see scales and instruments in Supplementary Material 2).

#### Data Analysis

In this study, a cross-sectional design was used with convenience samples mated by professional characteristics. Correlations, regressions, and mediational analyses were conducted amongst explanatory and explained variables of well-being, using SPSS 24 and process 3.4, Model 4. Scores were standardized and the correlations weighted by the inverse of the variance using the CMA program (Borenstein et al., 2014) for estimate a global effect size in this study.

## Results

To test H1, we examined the relations between well-being variables, using CMA to estimate a general effect size for the three samples. EPWB was associated with low BSCs (*r* = 0.46), IC 95% [0.41; 0.50], with QLLH (*r* = 0.52) IC 95% [0.48 to 0.56], lower negative AHWB (*r* = −0.35), IC 95% [−0.39; – 0.30] and higher positive AHWB (*r* = 0.45), IC 95% [0.40 to 0.49], as well as higher SWL (*r* = 0.58), IC 95% [0.55; 0.62]. Relations between variables were all > 0.70 (H1) (see Table 1)^6^. To test H2, relations between individual predictor variables were examined with well-being. EPWB is associated with greater EC (weighted correlation *r* = 0.14), IC 95% [0.09; 0.19]. This latter variable was also associated with lower QLLH (*r* = −0.062), with negative AHWB (*r* = 0.17) and more EPs (*r* = −0.12), although also with SWL (*r* = 0.087) and positive AHWB (*r* = 0.21), all of them *p* < 0.05 (H2a). EPWB was also associated with sharing OVC (*r* = 0.25) IC 95% [0.09; 0.19] and TVU (*r* = 0.23), IC 95% [0.19; 0.29]. Regarding H4, higher AM was associated with EPWB (*r* = 0.27, *p* = 0.0001) in the three samples. The greater the IS at the job position, the greater the well-being (*r* = 0.13, *p* = 0.001) in two of the three countries. On the other hand, SO was neither generally nor specifically associated with well-being in any of the countries. At microsocial level (H5), well-being was associated with CWa (*r* = 0.31), IC 95% [0.26; 0.36], with low EPs (*r* = −0.28), IC 95% [−0, 33; −0, 23] and high-quality LpB (*r* = 0.31), IC 95% [0.26; 0.36] (see by country on Table 2)^7^.

**TABLE 1:** Association between the variables that make up the nomological network of well-being at the individual level (Study 1).

Variables	*N*	*M*	*SD*	1	2	3	4	5	6
(1) BSCs	1298	45.20	8.18	–					
(2) QLLH	1268	53.36	9.23	0.65**	–				
(3) AHWB positive	1361	23.44	7.19	0.26**	0.40**	–			
(4) AHWB negative	1359	8.852	6.39	−0.50**	−0.49**	−0.26**	–		
(5) SWL	1334	38.53	6.64	0.34**	0.47**	0.40**	−0.33**	0.47**	
(6) EPWB	1352	81.33	13.74	0.42**	0.45**	0.41**	−0.31**	0.45**	0.55**

BSCs, behavioral, somatic and cognitive reactions to stress; QLLH, quality of life linked to health; AHWB, affective hedonic view of subjective Well-Being; SWL, cognitive hedonic view of subjective Well-Being or satisfactorial with life; EPWB, eudaimonic vision or psychological Well-Being and personal optimal development. ** *p* < 0.01.

**TABLE 2:** Relationship between individual and microsocial level predictor variables with psychological well-being by country (Study 1).

Variables	Chile				Spain				Uruguay			
	*n*	*M*	*SD*	*R*	*n*	*M*	*SD*	*r*	*n*	*M*	*SD*	*r*
Gender	342	1.66	0.473	0.03	284	1.79	0.415	−0.091^ߪ^	697	1.91	0.288	0.02
EC	352	64.98	13.07	0.07	289	62.1	9.96	0.13*	689	65.10	12.07	0.18**
OVC	355	28.32	4.951	0.18**	294	18.8	5.95	0.14**	689	26.84	4.924	0.29**
TVU	355	15.99	1.773	0.27**	296	7.69	4.48	0.23**	718	15.15	2.291	0.25**
SO	241	2010.2	7.643	–0.02	245	1985.9	180.7	0.04	649	2005.6	9.985	–0.04
AM	243	2.99	1.098	0.35**	264	3.56	1.003	0.25**	673	3.057	1.010	0.28**
IS	244	1.62	0.535	0.20**	260	1.75	0.537	–0.007	652	1.60	0.562	15**
EPs	361	12.80	4.307	−0.17**	294	12.4	3.24	−0.29**	716	13.88	3.505	−0.30**
CWa	360	26.46	6.798	0.23**	294	26.71	5.41	0.32**	697	26.45	5.403	0.35**
LpB	361	27.12	8.025	0.22**	280	28.26	5.70	0.36**	700	27.35	6.137	0.32**
EPWB	353	77.82	13.71	–	296	79.53	11.5	–	703	83.84	14.12	–

Gender = 1 = men; 2 = women; EC, emotional creativity; OVC, openness to change values; TVU, values of transcendence universalism; SO, seniority in the organization; AM, agreement with the methodology in the workplace; IS, intention to stay; EPs, excess of psychological demands at work or work stress; CWa, control over work, role autonomy; LpB, leadership that reinforces participation and belonging; EPWB, eudaimonic vision or psychological Well-Being and personal optimal development. ** *p* < 0.01, * *p* < 0.05, ^ߪ^p < 0.10.

To test H6, relations between meso-social-level predictor variables were examined with well-being. LeR, particularly the expression of negative emotions, neutral emotions, and emotional dissonance, were significant and negatively associated with all indicators and specifically with EPWB (*r* = −0.23, *r* = −0.10 and *r* = −0.17, respectively). The expression of positive emotions, although associated with AHWB, also did so with lower QLLH and greater negative AHWB. Control over interaction was positively associated with all indicators, just like sensitivity and empathy (except for QLLH and EPs). Examination of organizational culture showed that association of transformational culture with well-being is positive (*r* = 0.25), IC 95% [0.20; 0.30] and negative with transactional culture (*r* = −0.11), IC 95% [−0.16; −0.05] (see by country on Table 3)^7^ (H6).

**TABLE 3:** Relationship between mesosocial level predictor variables with well-being by country (Study 1).

Variables	Chile				Spain				Uruguay			
	*n*	*M*	*SD*	*r*	*n*	*M*	*SD*	*r*	*n*	*M*	*SD*	*r*
LeR	347	71.88	12.84	−0.11*	276	71.43	9.463	0.06	637	68.54	11.87	−0.13**
LeR NE	350	11.86	4.907	−0.21**	282	12.42	3.981	−0.11*	696	11.39	4.543	−0.26**
LeR NEE	349	10.67	3.131	–0.07	283	10.83	2.548	0.02	697	10.06	2.994	−0.11**
LeR PE	350	16.80	3.053	–0.04	284	16.11	2.951	0.16**	694	16.07	3.428	0.05
LeR ED	348	12.02	4.548	−0.11*	281	11.11	3.488	–0.08	687	11.69	4.300	−0.23**
LeR SandE	350	13.26	2.210	0.12*	284	13.58	1.692	0.15**	689	12.44	2.362	0.08*
LeR CINT	350	7.220	2.207	0.12**	282	7.361	1.750	0.27**	705	6.748	2.016	0.20**
CSO Transform.	350	2.25	9.108	0.10*	273	5.076	5.739	0.28**	650	4.181	6.742	0.26**
CSO Transacc.	348	0.160	5.575	–0.07	273	−0.2088	5.080	−0.25**	657	2.168	4.673	−0.17**

LeR, emotional labor role; LeR NE, negative expression of emotions in the work role; LeR NEE, neutral expression of emotions in the labor role; LeR PE, positive expression of emotions in labor role; LeR ED, emotional dissonance in the labor role; LeR SE, sensitivity and empathy in the labor role; LeR CINT, control of interaction in the labor role; CSO, culture and organizational structure that reinforces participation and integration (transformational and transactional). ** *p* < 0.01, * *p* < 0.05.

To examine the specific contribution of explanatory variables to well-being, we conducted a multiple regression of well-being (Hervás and Vázquez, 2013) on individual variables (EC and MV), on microsocial-level variables (CWa, EPs, and LpB) and meso-social or organizational variables (CSO). The multiple regression was significant, *F*(8,1121) = 30, 38, *p* = 0.0001, *R*^2^adjusted = 0.17. OVCs predicted well-being (β = 0.12, *p* = 0.002) along with TVUs (β = 0.09, *p* = 0.017), EC (β = 0.078, *p* = 0.007), low EPs (β standardized = −0.15, *p* = 0.0001), high CWa (β = 0.14, *p* = 0.0001), LpB (β = 0.10, *p* = 0.006) and transformational CSO (β = 0.09, *p* = 0.002). Only transactional culture did not obtain a significant coefficient (β = 0.004, *p* = 0.88) (see Table 4).

**TABLE 4:** Regression of well-being on individual, psychosocial and organizational culture factors (Study 1).

Variable	Model 1			Model 2			Model 3		
	*B*	*SE B*	*B*	*B*	*SE B*	*B*	*B*	*SE B*	*B*
EC	0.012	0.032	0.10***	0.011	0.032	0.10***	0.009	0.034	0.78***
OVC	0.026	0.085	0.12**	0.028	0.082	0.12**	0.027	0.008	0.12**
TVU	0.026	0.012	0.08*	0.025	0.012	0.08*	0.029	0.012	0.09*
EPs				–0.058	0.011	0.16***	–0.055	0.011	−0.15***
CWa				0.035	0.081	0.15***	0.034	0.084	0.14***
LpB				0.028	0.072	0.14***	0.022	0.080	0.10**
CSO transformational							0.017	0.006	0.09**
CSO transactional							0.003	0.008	0.010n.s
*R*^2^	0.052			0.017			0.018		
*F* for change in *R*^2^	24.140***			42.464***			30.138***		

CE, emotional creativity; OVC, openness to change values; TVU, values of transcendence, universalism; EPs, excess of psychological demands at work or work stress; CWa, control over work, role autonomy; LpB, leadership that reinforces participation and belonging; CSO, culture and organizational structure that reinforces participation and integration (transformational and transactional). * *p* < 0.05, ** *p* < 0.01, *** *p* < 0.001.

**FIGURE 1:**
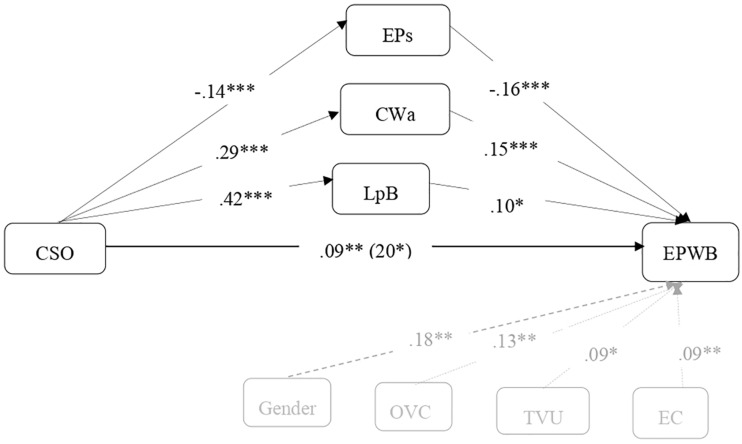
Effect of transformational organizational culture on psychological well-being and optimal personal development (EPWB), mediated by the psychosocial factors (EPs, excess of psychological demands at work or work stress; CWa, control over work, role autonomy; LpB, leadership that reinforces participation and belonging, group level). The model controls the values of openness to change (OVC), transcendence universalism (TVU) and emotional creativity (EC) related to the mediator and the variables of the result. Between brackets, total effect. All the values are standardized. ^∗^*p* < 0.05; ^∗∗^*p* < 0.01; ^∗∗∗^*p* < 0.001.

Using transformational CSO as a meso level predictor and controlling individual variables (gender, OVC, TVU and EC), a mediational analysis showed that the indirect effect of the CSO on the EPWB occurred through low EPs (β = 0.02), IC 95% [0.01; 0.04], as well as trough high levels of CWa (β = 0.04), IC 95% [0.02; 0.07] and LpB (β = 0.04), IC 95% [0.008; 0.07]. This effect explains 55% of the total effect (see Figure 1). The overall model was significant: *R*^2^ = 0.44, *F*(8,1101) = 32, 82, *p* < 0.001 (H6a).

### Gender Differences

We sought to discover the relation of gender with well-being indicators and possible differences between men and women. Regarding EPWB, no statistically significant differences were found in Chile or Uruguay [*r*_(__333__)_ = 0.03, *p* = 0.27, *r*_(__675__)_ = 0.02, *p* = 0.33], although they were found, to a lesser extent, in Spain [*r*_(__283__)_ = −0.091, *p* = 0.06]. Men report greater positive AHWB (*r* = −0.075, *p* = 0.006), although not homogeneously [Chile *r*_(__235__)_ = −0.14, *p* = 0.017, Spain *r*_(__240__)_ = −0.026 and Uruguay *r*_(__620__)_ = 0.02, *p* = 0.33]. No gender differences were found in negative AHWB in the three countries^8^ (H3).

## Discussion

In the general sample and by country, indicators of well-being were associated with one another, supporting H1 on the positive association between well-being indicators. Specifically, psychological well-being, was associated with having less stress and better quality of life, with life satisfaction and with affect balance. The strength of association between variables means that they are interrelated, yet they constitute independent constructs, in congruence with H1. As postulated by H2, emotional creativity, as well as the values of openness to change and transcending the self, were positively associated with well-being. Emotional creativity played an ambivalent role, associated both with well-being, and with psychological demands in the labor role, and lower quality of life in relation to health. Emotional creativity includes three dimensions: emotional preparedness, novel and authentic and useful emotional reactions. Studies show that is the dimension of novelty that is related positively to negative outcomes, while preparedness and effectiveness/authenticity are related to positives outcomes (Averill, 2009; Runco, 2014). These results suggest that attention and cognitive processing of intense emotion has “particular” effects on well-being and coping with stress. Probably because of the complexity and originality of creative emotions, different from schemas and cultural available scripts to describe them, high EC participants find difficulties with categorizing and identifying their emotions. Complexity and diversity of emotions also made difficult to elaborate linguistically and express affective state (Averill, 2009; Runco, 2014; Abuladze and Martskvishvili, 2016). Results confirm that certain values are individual factors of well-being. Openness to change (self-direction) and self-transcendence (universalism) are healthy values. They are cognitive representations of self-actualization or growth needs. Self-direction values and universalism are related to Deci and Ryan self-determination theory autonomy, competence and relatedness needs, respectively. Motivation to pursue healthy values enhances directly well-being because it satisfies intrinsic and self-actualizing needs. These healthy values afford well-being because they lead to positive and prosocial perceptions, attitudes and behaviors, like benevolent view of social world, trust in others and altruism (Schwartz and Sortheix, 2018).

Differences in well-being between genders were marginal, explaining maximum 1.9% of variance, in concordance with H3. Results support studies that had found greater hedonic affective well-being in men, and reject that women report more negative affectivity in these samples. Consistent with our hypothesis 4, agreement with the methodology at the workplace and the intention to stay long-term at the workplace are clearly associated with well-being. On the contrary, lower seniority was not associated with well-being, questioning this part of the hypothesis. It was argued that recent entry into an organization was associated with greater initial satisfaction (Romero, 2001). However, it is also possibly that people who stay long term in an organization can enhance control of their environment, obtain status and rewards, and consequently increase their satisfaction (Ronen, 1978). In particular, young teachers and educators could have a greater workload in more stressful settings, which may explain why the lower the seniority, the worse the perception of the social-emotional climate. Consistent with the H5, the results showed that high levels of control at work, social support and quality leadership, and low levels of psychological demands (Moncada et al., 2014) predicted well-being. Meso-social-level factors were also associated with well-being, in particular emotional labor, as H6 stated. This study confirmed that expressing and handling emotions as part of the labor role undermines well-being. In other words, having to express even positive emotions takes a toll, since this is associated with stress and negative affectivity, as well as lower perceived health. The importance of an organizational culture that facilitates social integration (Knight and Eisenkraft, 2015) was confirmed, because supporting H6 a transformational culture was associated with well-being. The opposite occurred with the transactional culture, which was associated with more stress, less control and less social and quality support. However, this result did not bear a significant multivariate coefficient, so the results relativize the negative nature for well-being of this type of culture. Along these lines, psychosocial factors were associated with a more transformational culture, mediating between culture and individual well-being, in concordance with H6a. To conclude, it should be stated that this study has limitations, the most noteworthy being that the sample was of convenience and that this is a correlational, not longitudinal or causal relationship study, which means that the results should be taken with caution.

### Study 2: Psychosocial Favorable Factors to Well-Being in Three Countries: The Case of a School With High SVI^9^ in Talca, Chile

This study analyzes the presence of risk factors for well-being in an educational organization with high school vulnerability^10^ (Ministerio de Desarrollo Social y Familia, 2020). Study 1 showed that well-being was linked to low stress, high control in the work role and social support – quality leadership low emotional work and an organizational culture and structure that most reinforces participation and integration in the organization. It also found medium-high level of autonomy, high stress and medium-low levels of social support and quality leadership of these sectors in the three countries (results not shown in this research). This second study explored whether these associations and well-being profile were replicated in the sample of educators and in particular in a Chilean school located in a difficult social context. The study of well-being in Chile, specifically in education, is a research priority (see López et al., 2017). Teachers’ well-being is relevant to the effectiveness of the teaching-learning process (Lever et al., 2017). Working conditions, both at the system and school level, can have an impact on teachers’ well-being (Viac and Fraser, 2020) and on students’ performance (Roorda et al., 2011; López et al., 2017).

This study aims to test the diagnostic capacity and relevance of the instruments used in the education sample in Chile, Spain, and Uruguay. As far as we know, this is the first comparative diagnosis, which is carried out on an education sample, in these three countries. Cut-off points of psychosocial factors are applied to scores of work stress; of control over work and to leadership that reinforces participation and belonging (which includes social support and quality leadership), emotional labor and culture and organizational structure that reinforces participation and integration (transformational culture) and individual well-being. Results are used to have a descriptive vision of the similarities between teachers from schools in the same locality and Country (Chile), students of Education from a private university (Chile), teachers from the region (Uruguay) and from a more culturally and economically distant area (Spain). We postulated that the profile would be similar in the Latin American samples (in Uruguay a large part of the schools in the sample could be categorized in “vulnerability”^11^), that a medium low psychosocial risk level would be found in the students of pedagogy of the private university and that the Spanish teachers would show the most positive psychosocial profile, due to the relative development of the country. In addition, it should be mentioned that the Spanish sample belongs to an educational center that develops a methodology that necessarily implies control over the tasks and team work.

This article also aims to confirm hypotheses 3–6 in the education sub-sample. That is, to know the specific association of individual well-being with gender (H3), low seniority in the organization; agreement with the methodology in the workplace and the intention to stay in your organization in the long term (H4); also with the psychosocial factors specified above (H5); as well as with meso-level variables such as the role of emotional labor and of the organizational culture that reinforces participation and integration. In this sense, we expected to confirm that a transformational organizational culture in schools is positively and more strongly associated with well-being than a transactional one (H6).

#### Sample

One thousand one hundred eighty-five individuals participated in this study. *n* = 37 [28 (75%)] women between 23 and 61 years of age (*M* = 39.14, *DT* = 10.23) kindergarten and primary education teachers at a public education establishment in the city of Talca, Chile (Group 1, high vulnerability school); *n* = 164 (123 (77%) women, between 19 and 66 years of age (*M* = 38.23, *DT* = 12,79) of educational establishments in the city of Talca, Osorno and Valparaiso, Chile (Group 2); *n* = 43 [26 (61%)] women, between 20 and 32 years of age (*M* = 23.35, *DT* = 2.51) student teachers from a private university -campus Talca, Chile- (Group 3) and *n* = 31 [7 (23%)] women, between 22 and 54 years of age (*M* = 24.93, *DT* = 5.91) student teachers from a private university –campus Santiago de Chile, Chile (Group 4); *n* = 212 [172 (86%)] women, between 25 and 60 years of age (*M* = 47.67, *DT* = 8.56) kindergarten and primary teachers in Public education establishments in the territories of Guipuzcoa, Alava, and Vizcaya (Group 5) and *n* = 699 [600 (86%)] women, between 22 and 69 years of age (*M* = 43.69, *DT* = 9.54) kindergarten and, to a greater extent, primary school teachers belonging to the country’s public institution of Montevideo in Uruguay (Group 6)^12^.

#### Procedure

This study was carried out within the framework of a general project developed in Chile, Spain and Uruguay in a sample of education and social intervention. In this case, teachers and university students of Pedagogy responded collectively and face to face during a period of 50–75 min a battery of instruments (only some of them are addressed in this study). The sample of Chilean teachers from Talca (Group 1) also answered the scale SusesoIstas21 (Candia et al., 2016). This instrument, which allows the assessment and measurement of psychosocial risks at work, is an adaptation of the Spaniard CoPsoQ-Istas21 Questionnaire (Moncada et al., 2014). The Chilean version of the instrument showed that in Group 1, 57% show high stress and low control of work; and almost 60% show low social support and quality leadership^13^. Profile found by the SusesoIstas21 in Group 1 (dimensions of stress, control of work, and social support and quality leadership) was replicated with CoPsoQ-Istas21 (dimension 1, 2, and 4). Although the profile was similar with both instruments, the SusesoIstas21 categorizes more people in risk scores (this is another study). Below, we examine the profile of the other samples to get an overview of the situation of teachers in the three countries.

#### Instruments

In this study, we addressed well-being (Diener, 1996; Fredrickson, 2013; Hervás and Vázquez, 2013)*;* psychosocial factors (Moncada et al., 2014), emotional labor role or LeR (Ortiz et al., 2012) and CSO (Nader et al., 2006) (for a more detailed version of the instruments see Study 1 and resource on line 2). Psychosocial factors were measured by some dimensions of CoPsoQ-istas21. This is an open access instrument of evaluation and prevention of psychosocial risks promoted by Scandinavian trade unions and researchers, which has been translated and validated by the CCOO trade union in Spain. CoPsoQ-Istas21 Questionnaire was applied and validated by the Sub-secretary of Economic and Social Affairs in Chile, creating a Chilean version called SusesoIstas21. In this study SO, AM and IS^14^ association with well-being and gender differences were also analyzed. Focus groups (Allen, 2017) were held, when possible, during the sessions to return the research results to the participating centers. We wanted to know the teachers’ perception of the results. The next step was to invite them to create working groups and plan short, medium – and long-term – actions based on the diagnosis of their school.

#### Data Analysis

Descriptive frequencies analyses were carried out. Tercile cut-off points were applied to the aforementioned sub-sample, comparing the results with paired samples of professors (Group 2) of the same zone (Chile) and the other two countries (Spain and Uruguay Groups 5 and 6 respectively) and upper-education students (Groups 2 from the same zone of the sub-sample and 3 from the capital of the country, Chile). Correlations were carried out, similar to those conducted in Study 1, but in this case using only the global teacher’s sample (Groups 2, 5, and 6) and the Group 1. Finally, mean comparisons were made between the Talca sample, supposedly the most negative context, and the Spanish sample, as the most positive context.

## Results

### Categorization of Responses According to Scales by Country and Sub-Sample

Compared with the sample of Chilean education and pedagogy students (groups 3 and 4) and with teachers from Spain and Uruguay (groups 5 and 6), the teachers from Chile (group2) and Talca (Group 1) display a medium-high level of Eps (group 6 reports a slightly higher level), lower CWa (the rest display a better profile), as well as low LpB in their organization (the others display a similar and better profile) (see Table 5). They also display relative lower AHWB and medium-high levels of EPWB. As previously mentioned teachers from Talca (group 1) displayed psychosocial risk levels above 50% in all dimensions of the equivalent Chilean questionnaire (see da Costa, 2018). Applying the cutoff points from the Spaniard scale (Moncada et al., 2014), around 9/10 subjects from this educational center display high EPs, 3/10 low CWa and 6/10 low LpB quality.

**TABLE 5:** Psychosocial risk level, EPWB, AHWB, LeR affective and CSO that reinforces participation and integration (Study 2).

	Teachers		Students Pedagogy chilenos		Teachers	
	Group 1 Chileans in the South Central Region^1^	Group 2 Other teachers South Center^2^	Group 3 South Central Region^3^	Group 4 Metropolitan region^4^	Group 5 ACBC Spain^5^	Group 6 Uruguay^6^
**EPs (Moncada et al., 2014)**						
Low risk (0–7)	8.1%	10.2%	7%	12.9%	3,8%	4%
Medium risk (8–11)	16.2%	20.5%	20.9%	41.9%	34%	20.3%
High risk (12 or more)	75.7%	69.3%	72.1%	45.2%	62.2%	75.7%
**CWa (Moncada et al., 2014)**						
Low risk (0–18)	30.6%	16.3%	16.3%	6.5%	5.8%	6.9%
Medium risk (19–25)	38.9%	24.1%	16.3%	6.5%	37.5%	36.1%
High risk (26 or more)	30.6%	59.6%	67.4%	87.1%	56.7%	57%
**LpB (Moncada et al., 2014)**						
Low risk (0–24)	50%	35.5%	23.3%	20%	23.3%	30.3%
Medium risk (25–31)	36.1%	38%	51.2%	4,4%	53.4%	49%
High risk (32–40)	13.9%	26.5%	25.6%	76%	23.3%	20.8%
**EPWB (Hervás and Vázquez, 2013)**						
Low Well-Being (74 and less)	50%	41.5%	44.2%	53.3%	36%	24.2%
Medium risk (75–83)	41.7%	23.8%	32.6%	30%	32.2%	16.9%
High Well-Being (84 or more)	8.3%	34.8%	23.3%	16.7%	31.8%	58.9%
**AHWB (Fredrickson, 2013)**						
Negative Balance (0 or less)	18.9%	5.4%	7%	11%	9%	14.5%
Slightly positive balance (1–10)	29.7%	16.7%	16.3%	11%	15.7%	19.8%
Positive balance (11 or more)	51.4%	78%	76.7%	78%	75.2%	65.7%
**LeR (Ortiz et al., 2012)**						
Low levels of empathy and sensitivity (8 or less)	2.8%	3.1%	2.3%	0%	1.9%	5.4%
Medium level of empathy and sensitivity (9–13)	41.7%	31.4%	44.2%	50%	34.6%	57%
High level of empathy and sensitivity (14 or more)	55.6%	65.4%	53.5%	50%	63.5%	37.6%
Low expression of negative emotionality (8 or less)	30.6%	35.2%	14%	6.7%	14.4%	23.9%
Medium level of negative emotional expression (9–10)	16.7%	20.8%	16.3%	16./%	16.7%	17%
High level of negative emotional expression (11or more)	52.8%	44%	69.8%	76.7%	68.9%	50.1%
Low levels of emotional dissonance (10 or less)	40%	44.7%	25.6%	23.3%	47.6%	37.1%
Medium levels of emotional dissonance (11 to 15)	40%	35.8%	25.6%	46.7%	43.8%	41%
High levels of emotional dissonance (16 or more)	20%	19.5%	48.8%	30%	8.7%	21.9%
**CSO (Nader et al., 2006)**						
Low transformational CSO (− 6 and less)	54.3%	14.8%	11.6%	6.7%	9.8%	9.8%
Medium level of transformational CSO (−5 and 5)	37.1%	22.2%	23.3%	30%	32.6%	36.2%
High level of transformational CSO (6 or more)	8.6%	63%	65.1%	63.3%	57.5%	54%
Low transactional CSO (−6 and less)	45.7%	15.4%	9.3%	0%	12.4%	7.4%
Medium level of transactional CSO (−5 and 5)	48.6%	57.4%	46.5%	63.3%	68.1%	62,7%
High transactional CSO (6 o más)	6.7%	27.2%	44.2%	43.3%	19.5%	30%

*Sub-sample analyzed Group ^1^*n* = 37 (Talca); Group ^2^*n* = 164 (Valparaíso, Talca and Osorno); Group ^3^*n* = 43 (Talca)4 ^4^*n* = 31 (Santiago de Chile), Group 5 ^5^*n* = 212 (Guipuzcoa and Bizkaia); Group 6 ^6^*n* = 699 (Uruguay). EPs, excess of psychological demands at work or work stress; CWa, control over work, role autonomy; LpB, leadership that reinforces participation and belonging; EPWB, eudaimonic vision or psychological well-being and personal optimal development; AHWB, affective hedonic view of subjective well-being; LeR, emotional labor role, LeR SE, sensitivity and empathy in the labor role; LeR NE, negative expression of emotions in the labor role; LeR ED, emotional dissonance in the labor role; CSO, culture and organizational structure that reinforces participation and integration (transformational and transactional).*

Regarding LeR a similar profile was found between the samples. The school in Talca reports that almost 54% of the members who participated in this study report high expression of negative emotions as part of their role (versus 44% of group 2, 69% of group 5 and 50% of group 6), although did not report high emotional dissonance (20% versus 20% of group 2, 9% of group 5 and 22% of group 6). Regarding the organizational culture, and broadly distanced from the other samples, this educational establishment perceives low levels of transformational CSO in its organization, in addition to medium-low levels of transactional CSO.

To examine the specific hypothesis of the negative profile of Talca teachers by respect to Spanish teachers, means were compared between the Group 1sample and the Spanish sample with a paired *t*, using the *dt* from the first sample for the contrast. Chilean teachers (Group 1) displayed greater EPs (*M* = 14.27 vs. *M* = 13.14, *t* = 2.14, *p* < 0.04), lower CWa (*M* = 22.5 vs. *M* = 26.2, *t* = 4.24, *p* < 0.001) and LpB (*M* = 23.36 vs. *A* = 28.16, *t* = 4.22, *p* < 0.001) than Spanish teachers. Regarding BH, teachers from Group 1 displayed a similar level of positive affectivity as in the comparison sample (*M* = 21.8 vs. *M* = 22.35, *t* = n.s). On the other hand, Chilean teachers display greater negative affectivity (*M* = 12 vs. *M* = 7, *t* = 4.7, *p* < 0.001). The EPWB of Chilean teachers from Talca was lower than their counterparts in Spain (*M* = 71.5 vs. *M* = 79.45, *t* = 3.45, *p* = 0.001).

#### Gender Differences

Regarding H3, unifying the sample of teachers from Chile (Groups 1 and 2), men show a higher EPs profile than women, although the score is marginal (*M* = 12.89 vs. *M* = 12.54, *t* = 0.18, *p* < 0.07). They also show greater emotional dissonance *r*_(__189__)_ = −0.14, *p* = 0.02 (*M* = 12.86 vs. *M* = 11.35, *t* = 0.20, *p* < 0.05). No statistically significant differences in these variables are found in Group 1. In this group, men show greater social support and quality leadership *r*_(__35__)_ = −0.34, *p* < 0.02 (*M* = 28.22 vs. *M* = 22.80, *t* = 0.21, *p* < 0.046), empathy and sensitivity *r*(35) = −0.39, *p* < 0.009 (*M* = 14.62 vs. *M* = 12.66, *t* = 0.25, *p* < 0.02) as well as control of interaction in the work role *r*_(__35__)_ = −0.43, *p* < 0.005 (*M* = 8.50 vs. *M* = 6.44, *t* = 0.28, *p* < 0.009). Female teachers from the Spanish sample show greater expression of positive emotions in the work role *r*_(__198__)_ = 0.23, *p* = 0.001 (*M* = 16.29 vs. *M* = 14. 28, *t* = −3.08, *p* = 0.02) as well as greater empathy and sensitivity in the work role *r*_(__198__)_ = 0.24, *p* = 0.001 (*M* = 13.78 vs. *M* = 12.61, *t* = −3.30, *p* = 0.001). Finally, no statistically significant differences are found in Uruguay except in EPs, where female teachers show a somewhat higher score *r*_(__675__)_ = 0.06, *p* < 0.05 (*M* = 13.83 vs. *M* = 13.13, *t* = −0.17, *p* < 0.09).

### Psychosocial Risks, Emotional Work Role Factors, Organizational Culture, and Their Relationship With Well-Being Indicators

Psychosocial risks factors and organizational culture are associated with well-being with a profile similar to Study 1 in the global sample of educators in concordance with H4, 5 and 6^15^. In Talca sub-sample (Group 1) agreement with the methodology used in their workplace correlated with affect balance and psychological well-being (see Table 6) (H4). EPs is negatively correlated with AHWB and e affect balance, and marginally so with SWL. CWa is positively and significantly associated to AHWB (negatively with negative affectivity), SWL and also to EPWB (H5). Empathy and sensitivity as a demand of the labor role held by educators display a similar profile. The emotional labor of expressing negative emotions in the work role was positively and significantly associated with negative affectivity, and negatively with SWL. Finally, emotional dissonance was associated, to a lesser extent and negatively, with positive affectivity and balance of affect. Correlation with negative AHWB is positive and marginal. The relation between the indicators of well-being and CSO in this sample were n.s. (H6).

**TABLE 6:** Association between individual, micro and mesosocial level variables and well-being in education sample (Study 2).

	Chilean, Spanish, and Uruguay teachers									Chile, Talca								
	*N*	1	2	3	4	5	6		7	*n*	1	2	3	4	5	6		7
							T^1^	S^2^								T^2^	S^3^	
Gender	1000	–0.02	−0.08**	−0.07*	–0.007	–0.04	–0.005	–0.05	0.10**	36	–0.07	0.06	–0.03	–0.10	0.04	−18	0.13	0.06
EPs	1030	−0.41**	−0.43**	−0.31**	0.39**	−0.44**	−0.33**	−0.22**	−0.23**	36	0.05	0.007	−0.28*	0.32*	−0.35*	−0.23^ߪ^	–0.16	–0.20
CWa	1020	0.17**	0.29**	0.38**	−0.22**	0.39**	0.41**	0.24**	0.29**	36	–0.21	−0.23^ߪ^	0.32*	–0.17	0.29*	0.54**	0.36*	0.34*
LeR	960	−0.21**	−0.21**	−0.08**	0.22**	−0.19**	−0.11**	–0.05	−0.10**	34	0.03	−19	0.007	0.27^ߪ^	–0.15	0.39*	0.12	0.12
LeR NE	1020	−0.23**	−0.26**	−0.22**	0.25**	−0.30**	−0.21**	−0.17**	−0.21**	36	0.03	−0.26^ߪ^	0.003	0.30*	–0.16	0.32*	0.29*	0.15
LeR NEE	1020	−0.14**	−0.15**	–0.06	0.17**	−0.14**	−0.08**	–0.03	−0.10**	35	0.05	0.11	–0.08	0.09	–0.10	0.32*	0.21	0.23^ߪ^
LeR PE	1020	−0.06*	−0.07*	0.06*	0.06*	–0.006	–0.006	0.09**	0.05	36	0.07	0.007	0.02	–0.15	0.09	–0.008	–0.003	–0.10
LeR ED	1010	−0.27**	−0.26**	−0.16**	0.29**	−0.28**	−0.18**	−0.12**	−0.15**	35	–0.09	–0.03	−0.27^ߪ^	0.26^ߪ^	-0.31*	0.02	–0.07	–0.10
LeR SandE	1010	0.03	0.04	0.11**	–0.04	0.09**	0.09**	0.07*	0.03	36	0.12	0.05	0.40**	–0.16	0.33*	0.51**	0.06	0.37*
ReL CINT	1025	0.13**	0.14**	0.22**	−0.18**	0.25**	0.19**	0.12**	0.13**	36	–0.18	−0.25^ߪ^	0.23^ߪ^	0.19	0.04	0.23^ߪ^	–0.13	0.12
LpB	1020	0.23**	0.31**	0.33**	−0.31**	0.41**	0.37**	0.22**	0.32**	36	–0.11	0.06	0.27^ߪ^	–0.21	0.28*	0.37*	0.12	0.31*
CSO Transform.	960	0.13**	0.16**	0.24**	−0.18**	0.27**	0.21**	0.10**	0.21**	35	−0.24^ߪ^	–0.04	–0.05	0.29*	–0.19	–0.12	−0.29*	–0.12
CSO Transacc.	960	−0.21**	−22**	−0.14**	0.28**	−0.26**	−0.18**	−0.07*	−0.08**	35	–0.05	–0.11	0.17	–0.22	0.22	0.31*	0.07	0.23^ߪ^
SO	850	0.004	–0.02	–0.003	–0.009	0.003	–0.02	0.006	0.01	36	0.006	–0.19	–0.04	0.19	–0.13	–0.07	–0.02	–0.15
AM	890	0.29**	0.32**	0.27**	−0.24**	0.32**	0.34**	0.21**	0.29**	36	0.09	–0.05	0.18	–0.31	0.28*	0.53**	0.22^ߪ^	0.39**
IS	890	0.15**	0.14**	0.16**	−0.22**	0.24**	0.26**	0.07*	0.13**	10	–0.03	–0.03	0.83**	0.47^ߪ^	0.24	0.64*	0.37	0.36

1 = BSCs (behavioral, somatic and cognitive reactions to stress); 2 = QLLH (quality of life linked to health); 3 = AHWB positive (affective hedonic view of subjective Well-Being); 4 = AHWB negative (affective hedonic view of subjective Well-Being); 5 = affect balance; 6 = SWL (cognitive hedonic view of subjective Well-Being or satisfaction with life), ^1^Job satisfaction; ^2^Satisfaction with yourself; 7 = EPWB (eudaimonic vision or psychological Well-Being and personal optimal development). Gender = 1 = women, 2 = men. EPs, excess of psychological demands at work or work stress; CWa, control over work, role autonomy; LeR, emotional labor role; LeR NE, negative expression of emotions in the labor role; LeR NEE, neutral expression of emotions in the labor role; LeR PE, positive expression of emotions in the labor role; LeR ED, emotional dissonance in the labor role; LeR SE, sensitivity and empathy in the labor role; LeR CINT, control of interaction in the labor role; LpB, leadership that reinforces participation and belonging; CSO, culture and organizational structure that reinforces participation and integration (transformational and transactional); SO, seniority in the organization; AM, agreement with the methodology in the workplace; IS, intention to stay. ** *p* < 0.01, * *p* < 0.05, ^ߪ^*p* < 0.10.

## Discussion

Regarding the first objective of this study, to have a descriptive vision of the similarities of teachers in different nations and context, the general profile of the educators from the three countries shows that participants perceive high levels of psychological stress, low-medium levels of social support and quality leadership, high levels of demand to express negative emotions (although not emotional dissonance), medium-high levels of work control and of transformational culture in their organizations. The profile of the first three variables is one of risk, and it also shows that emotional dissonance is not a dominant element in these samples, but rather having to express negative emotions to control students (according to statements made by participants interviewed in focus group). Social support (Turner and Turner, 2013), which is at medium-low level with the majority of the general sample, is associated with psychological adjustment (Bender et al., 2019) and with greater life satisfaction (Chu et al., 2010). Its absence can be a clear symptom of lack of well-being. On the other hand, work control was at medium-high levels in most groups. This might explain the medium-high and high profile of psychological and hedonic well-being of teachers in the three countries. This suggests that resilience processes are present in the educators participating in this study.

Regarding to second objective, results confirm that psychosocial risk factors are higher in the sample of Chilean teachers than in the Spain sample. Two of the environmental psychological and social characteristics that facilitate well-being, such as low work-load and stress, support from peers and quality leadership are absent or at a low level in the Talca sub-sample. Moreover, educators from Talca at the analyzed educational establishment displayed risk levels in the following variables: psychological demands or stress, control of work or autonomy, social support and quality leadership, compensation, and double presence, measured with SusesoIstas21 (data not shown). Talca teachers also perceives low levels of transformational CSO in its organization, in addition to medium-low levels of transactional CSO. This deficient organizational culture profile must be understood within the framework of Chilean institutional operation. The difficulty in planning to make structural changes to management at this educational establishment was mentioned, as it is under the Municipal Department of Education, and because of the rigidity of its transactional culture. The people at this establishment perceive the absence of transformational culture. On the other hand, the context wherein the school operates must be considered, where students are very vulnerable socially with socioeconomic fragility and intra-family violence, leading to greater demands placed on teachers. However, professionals in the counseling department raise the possibility of intervening at an individual and group level in order to contribute to improving the well-being of these educators.

In concordance with H3 gender differences were not strong and heteroclite. Contrary to what was expected (Kenworthy et al., 2014), in the total teachers’ sample women report higher EPWB. And men report higher stress and emotional dissonance than women in the global Chilean sample. The opposite was found in Uruguay where women score higher on EPs as expected (Galanakis et al., 2009). One possible explanation is that primary household responsibilities (Sharma et al., 2016; Chawla and Sharma, 2019) have a greater interference with women’s work. In contrast, due to job or employment loyalty, job interference would be less for men. Studies also report that differences may be between cohorts rather than in gender *per se* (Galanakis et al., 2009). Recall that the meta-analysis by Purvanova and Muros (2010) found that while women were more emotionally burned than men, men showed slightly more depersonalization than women. The meta-analysis by Kenworthy et al. (2014) linked emotional dissonance to burnout, finding that employees who faked their emotions at work also suffered from burnout. In this study, the relationship between excessive psychological demands at work or work stress and emotional dissonance was positive and significant for the sample of Chilean teachers (results not shown). Accumulated evidence indicates that these risk factors for well-being can be addressed through interventions (e.g., Galanakis et al., 2009; Mesmer-Magnus et al., 2012; Bolier et al., 2013; Chaplin and Aldao, 2013; Lever et al., 2017; Slemp et al., 2018; Mendoza-Castejon et al., 2020; Pyhältö et al., 2020), with a gender perspective (e.g., Shannon et al., 2019). In the group 1 men report high positive outcomes in emotional labor as well as higher quality leadership and support. The opposite was found in Spain where women showed higher scores in positive emotion expression, empathy and sensitivity in the work role. This last data is consistent with research that highlights the relevance of contextual factors in gender differences (Chaplin and Aldao, 2013; Olson et al., 2019). Considering that providing support for autonomy can be a practical leadership approach to fostering the satisfaction of basic needs, the internalization of work motivation, and positive work outcomes (Slemp et al., 2018), one might ask what leads the men in this Talca school (Group 1) to perceive more social support and quality leadership than their female counterparts? Moreover, the enormous accumulated evidence on leadership, social support and its well-being benefits show that it is also possible to intervene on this point (e.g., Kossek et al., 2011; Eby et al., 2013; Carreira, 2017; Mathieu et al., 2019; Tifferet, 2020).

AM and IS correlate with well-being in the global teacher sample, in line with H4. H5 was also supported: low EPs, high CWa and LpB were related to EPWB. Regarding to H6, LeR, NE, NEE, and ED, as well as transformational culture, were negatively related to EPWB. Profile of correlations were similar for Talca subsample, but only AM, CWa, and LpB associations were significant – probably because of the small sample size. As stated, one of this study’s clear limitations is the size of the Talca sample. However, the results of the general education sample have conclusively confirmed hypotheses 4, 5, and 6. However, the results also suggest that the psychological disposition of the professionals toward their work and their high involvement with the organization, possibly influenced by the “vocational” character toward teaching, contributes as “a kind of palliative or compensatory mechanism” to the difficulties, risks or deficiencies that can be found in groups, organizations and systems that nestle them. At present, the educational center in Talca has started to carry out self-care activities at the end of each semester. This support consists of taking active breaks, carrying out reflection activities sent by the Ministry of Education and taking a walk at the end of the year. However, the feasibility and effectiveness of these organizational improvements remains open. Decision makers should be reminded of the relevance of teachers to the community and thus contribute to its well-being (Roorda et al., 2011; López et al., 2017; Viac and Fraser, 2020).

### Study 3: Favorable Factors to Well-Being in Five Countries: Labor and Military Organizations

This study involves military cadets from a higher education organization and workers from five countries and different employment contexts (including higher education organizations). With the sample of workers (WS) from two countries, the aim was to contrast the positive relationship between individual well-being (QLLH or health-related quality of life) and collective well-being (GHWB or socio-emotional climate), which in this study takes center stage as an explained variable (H1). We also sought to test hypotheses 2 to 6, that is, to check the positive association of collective well-being (GHWB) with the values of openness to change (OVC) and transcendence (TV) in a military sample (MS) (H2); gender differences in all samples (H3); as well as the positive correlation of collective well-being (GHWB) with seniority in the organization (SO), intention to stay (IS) in WS and the degree of knowledge and previous participation in work teams (KPW) in WS and MS (H4). We also seek to contrast (in MS and WS) the positive relationship of collective well-being (GHWB) with micro-social factors such as group participation and internal communication (IPaCG), task orientation and climate of excellence (TOaCE); the leadership style that contributes to reinforce social integration and participation (LpB) in MS and the negative relationship between collective well-being and somatic and cognitive reactions to stress (BSCs) in WS. Regarding leadership style, we think that the relationship would be stronger between collective well-being and transformational than with transactional leadership style (H5). At a mesosocial level we tried to contrast that labor role (LR) was positively associated with both individual (QLLH in WS) and collective well-being (GHWB in MS and WS); that both organizational leadership (LpO) and the culture and structure that reinforce participation and integration in the organization (CSO) are positively associated with collective well-being (GHWB). With regard to culture, we believe that the relationship would be stronger between collective well-being and the style of transformational versus transactional organizational culture (H6). Finally, it was postulated that the positive relationship between TOaCE and GHWB would be moderated by organizational leadership that reinforces organizational participation and integration (MS and WS). It was also postulated that gender would modulate the relationship between TOaCE and GHWB, as well as the relationship between LpO and GHWB. Military culture is characterized by strong values of masculinity in terms of competence, and toughness (Hofstede, 1998 in da Costa, 2018). Given the military’s organizational culture, it is expected that the functional processes within it will be more beneficial to male than female cadet, as postulated by H6b.

## Method

### Sample

Participating in this study were *N* = 1078 individuals (63% men), workers with a professional contract (72%) and cadets from a military school (28%). They reported being between 18 and 75 years old (*M* = 31.73, *SD* = 10.63), and being residents in Latin America countries (81.4%–86.5% birthplace) and Southern Europe (14.2%–12.5%), 78.7% had tertiary studies^16^.

### Procedure

Workers could respond on paper or online for a period of between 50 and 75 min, to different instruments. This study only addresses some of them. Completion was supervised by researchers or staff trained to this end in each one of the participating countries. Students’ data was collected for a week at the Military Institute and was supervised by a team of researchers trained for the task. Both projects shared the group-level DV in this study.

### Instruments^17^

Individual well-being was measured as QLLH, is measured through 12 items (general and mental health and vitality). These were selected from the instrument by Ware and Sherbourne (1992). A *Likert-like* scale with 5 anchor points was used for the first dimension (totally true = 1 to 5 = totally false) and 6 anchor points for the other two (always = 1 to 6 = never). Cronbach’s α: 0.80 for the three dimensions. The BSCs were measured as described in Study 1 (Setterlind, 2001 in Moncada et al., 2014). Both instruments were only used with workers from Spain and Argentina. GHWB is measured through the socio-emotional climate dimension by da Costa et al. (2016) (α = 0.86), along with the dimension of cohesion as a group process (α = 0.88) from the same instrument for both samples (*r* = 0.75). A *Likert* scale with 7 anchor points is used (not applicable at all = 1 to 7 = highly applicable). Cronbach’s α: 0.90. The MV are measured in this study with the same instrument as Study 1 (Schwartz, 2001) and 11 items. Cronbach’s α: 0.69 (OVC) and 0.71 (self-transcendence: TVB and TVU). KPW is measured with 4 items (da Costa et al., 2016) using a dichotomous scale (1 = no, 2 = yes). Spearman Brown: 0.70. IPaCG, as well as TOaCE, are respectively evaluated with 3 items from group processes (da Costa et al., 2016) and 7 anchor points (not applicable at all = 1 to 7 = highly applicable). Cronbach’s α: 0.84 and 0.86. LR is measured with 6 items and 7 anchor points (da Costa et al., 2016). Cronbach’s α: 0.85 (complexity and challenge of the role) and 0.62 (autonomy) *r* = 0.69. Cronbach’s α: 0.87. CSO is measured with 7 items and 7 anchor points (da Costa et al., 2016). Cronbach’s α: 0.88 (organizational integration) and 0.81 (resources) *r* = 0.74, Cronbach’s α: 0.90. LpB is evaluated with 34 items and 5 anchor points (totally disagree = 1 to 5 = totally agree). The perceived leadership in superiors (LpB) in the military setting is assessed (adapted from Castro Solano et al. in Nader and Sánchez, 2010). Finally, LpO is evaluated with 4 items of positive leadership in the organization, using the aforementioned scale (da Costa et al., 2016) with 7 anchor points. Cronbach’s α: 0.91. Gender, SO and the IS as the type of relationship had with their organization (1 = permanent, 2 = temporary, 3 = self-employed) are also analyzed in this study^18^.

### Data Analysis

Correlations, regressions, and moderation were analyzed with Model 1 from Process 3.4. The CMA program, version 3, was applied (Borenstein et al., 2014) to analyze correlations for different samples and obtain a weighted average effect size. Associations are shown separately on Table 7 and all together beginning with Table 8.

**TABLE 7:** Relationship between individual and microsocial level predictor variables with psychological well-being (Study3).

Variables	WS				MS			
	*n*	*M*	*SD*	*r*	*n*	*M*	*SD*	*r*
Gender	613	1.49	0.500	0.06^ߪ^	308	1.12	0.329	–0.06
SO	562	4.101	1.384	−0.16**	–	–	–	–
KPW experience	684	2.915	0.8884	0.10**	301	3.000	0.7023	0.12*
KPW training	681	3.180	0.7304	0.13**	307	3.130	0.7514	11*
IS	552	0.1920	10.42	0.03	–	–	–	–
OVC	–	–	–	–	308	27.50	4.614	–0.03
TVU	–	–	–	–	310	24.98	3.809	–0.02
IPaCG	768	13.54	4.815	0.74**	310	13.08	4.458	0.63**
TOaCE	749	14.62	4.200	0.76**	309	14.03	4.489	0.69**
LpB (transformational)	–	–	–	–	290	54.11	18.143	0.12*
LpB (transactional)	–	–	–	–	302	31.68	7.917	09^ߪ^
LR	687	27.83	7.664	0.71**	301	27.28	7.805	0.69**
CSO	688	25.26	8.453	0.73**	305	22.97	8.564	0.64**
LpO	703	37.67	11.85	0.78**	284	34.26	12.35	0.66**
GHWB	683	28.16	8.590	–	305	27.05	7.504	–

Gender = 1 = women, 2 = men; SO, seniority in the organization; KPW, degree of knowledge and previous participation in work teams; IS, intention to stay; OVC, openness to change values; TVU, values of transcendence universalism; IPaCG, internal participation and communication in the group; TOaCE, task orientation, and climate of excellence; LpB, leadership that reinforces participation and belonging (transformational and transactional); AR, labor role; CSO, culture and organizational structure that reinforces participation and integration; LpO, organizational leadership that reinforces participation and belonging; GHWB, group hedonic Well-Being. ** *p* < 0.01, * *p* < 0.05, ^ߪ^*p* < 0.10.

**TABLE 8:** Relationship between individual and microsocial level predictor variables with psychological well-being by country (Study 3).

Variables	WS					
	*n*	Mean	*SD*	*r*^1^	*r*^2^	*r*^3^
**Argentina**						
Gender	50	1.34	0.479	0.11	0.23	0.30*
SO	50	3.500	1.373	–0.10	–0.19	−0.41**
KPW	50	4.900	1.199	–0.22	0.16	0.32*
IS	50	1.240	0.5554	0.04	0.11	0.13
IPaCG	50	14.66	3.172	0.29*	–0.04	0.28*
TOaCE	50	15.70	2.589	0.07	–0.08	0.35**
LR	50	29.72	4.965	0.35**	0.09	0.01
CSO	50	28.10	5.761	0.40**	0.24*	0.47**
LpO	50	43.84	5.686	0.42**	0.14	0.28*
BSCs^1^	50	24.28	7.214	–	0.46**	0.19^ߪ^
QLLH^2^	50	44.10	4.418	–	–	0.34**
GHWB^3^	50	31.66	4.706	–	–	–
**Brazil**						
Gender	242	1.60	0.490	–	–	0.08
SO	248	4.076	1.306	–	–	–0.04
KPW	270	6.596	1.233	–	–	14*
IS	247	1.295	0.6029	–	–	0.18**
IPaCG	308	13.73	4.894	–	–	0.72**
TOaCG	299	14.71	4.313	–	–	0.74**
LR	275	27.60	7.797	–	–	0.73**
CSO	275	25.82	8.762	–	–	0.74**
LpO	288	38.31	11.81	–	–	0.78**
GHWB	273	28.64	8.282	–	–	–
**Southern Europe**						
Gender	153	1.46	0.500	−0.21*	−0.31**	−0.12^ߪ^
SO	100	4.840	1.488	0.02	0.08	−0.16^ߪ^
KPW	189	5.862	1.280	0.06	0.25**	0.14*
IS	82	1.426	0.5885	–0.15	–0.13	0.12
IPaCG	239	13.44	4.736	0.12	0.17*	0.73**
TOaCG	228	14.36	4.186	0.07	0.10	0.76**
LR	191	28.00	7.331	0.03	0.11	0.66**
CSO	191	24.42	8.447	0.007	0.07	0.69**
LpO	206	35.88	12.11	–0.02	0.04	0.74**
BSCs^1^	101	47.90	7.400	–	0.74**	0.17*
QLLH^2^	101	44.43	6.973	–	–	0.26**
GHWB^3^	193	27.18	9.043	–	–	–
**Mexico**						
Gender	133	1.40	0.491	–	–	0.24**
SO	133	3.646	1.142	–	–	–0.14
KPW	133	5.812	1.547	–	–	0.15
IS	133	0.3083	12.24	–	–	0.06
IPaCG	132	13.25	4.981	–	–	0.79**
TOaCG	133	14.55	4.423	–	–	0.86**
LR	132	27.56	8.647	–	–	0.84**
CSO	133	24.71	8.239	–	–	0.75**
LpO	129	37.22	11.99	–	–	0.86**
GHWB	128	28.14	8.980	–	–	.
**Latin America**						
Gender	441	1.51	0.501	–	–	0.12**
SO	447	3.892	1.287	–	–	−0.09*
KPW	469	6.179	1.432	–	–	0.12**
IS	446	0.8027	6.728	–	–	0.05
IPaCG	505	13.74	4.770	–	–	73**
TOaCG	498	14.84	4.174	–	–	0.76**
LR	473	27.93	7.775	–	–	0.74**
CSO	474	25.82	8.357	–	–	0.73**
LpO	480	38.66	11.51	–	–	0.79**
GHWB	467	28.87	8.184	–	–	–

The data for Chile is only presented in the section on Latin America as it is a very small sample of experts (*n* = 15). SO, seniority in the organization; KPW, degree of knowledge and previous participation in work teams; IS, intention to stay; IPaCG, internal participation and communication in the group; TOaCG, task orientation, and climate of excellence; LR, labor role; CSO, culture and organizational structure that reinforces participation and integration; LpO, organizational leadership that reinforces participation and belonging; BSCs, behavioral, somatic and cognitive reactions to stress; QLLH, quality of life linked to health; GHWB, group hedonic Well-Being; r^1^, r^2^, and r^3^, relationship of the variable with BSCs^1^, QLLH^2^ and GHWB^3^. ** *p* < 0.01, * *p* < 0.05.

## Results

To test the first hypothesis (H1) in this study, Argentina’s and Spain WS was used. The relationship between individual well-being (QLLH) and group (GHWB) well-being was analyzed with CMA. The average weighted association with the random model was *r* = 0.29, IC 95% [0.08; 0.49]. The heterogeneity test was not significant, *Q* = 1.77, *p* = 0.18. To test H2, a correlational analysis in the MS between OVC, TV and GHWB was conducted. The association was n.s. Regarding H4, a significant association was found between SO, with GHWB [*r*_(__562__)_ = −16, *p* < 0.001]; however, the relation with IS was n.s. The relation between KPW [*r*_(__968__)_ = 0.13, *p* < 0.001] with GHWB was significant, both in WS and in MS (see Tables 7, 8). Regarding H5 both in WS and in MS, IPaCG (*r* = 0.74 y 0.63) and TOaCE (*r* = 0.76 y 0.69) were respectively associated with GHWB in the organization (see Table 7 for general sample). Concerning to H6, GHWB was associated with the perception of LR (*r* = 0.62), all *p* = 0.001.

With the objective of testing H5 with a personal well-being indicator, perceptions of group processes (PyGG and TOaCE) were analyzed with QLLH in Argentina and Spain (WS). The values found were n.s. In the sub-sample (or sample from Argentina and Spain), the negative relation between stress and individual and collective well-being hypothesized by H5 was confirmed: BSCs was negatively associated with individual well-being: using the random model, mean weighted association of *r* = 0.50, IC 95% [0.38; 0.61] was found significant for both countries. The heterogeneity test was not significant *Q* = 0.21, *p* = 0.66. Low BSCs were associated with GHWB, the average weighted correlation using the same model was *r* = 0.19, IC 95% [0.04; 0.33]. The heterogeneity test was not significant *Q* = 0.405, *p* = 0.50. Finally, the relation of the transformational LpB was analyzed with GHWB (MS). A positive association was found (see Table 8), specifically with the individual level transformational scale [*r*_(__297__)_ = 0.15, *p* < 0.005]. Regarding H6, the relation between GHWB and LR proved to be significant *r* = 0.71, (see Table 7) both in WS and in MS. The association of individual well-being (QLLH), with role complexity in the Argentinean-Spanish sub-sample was n.s.

The perception of an integrative organization that invests resources to adequately conduct work was associated with GHWB *r* = 0.68 (see Table 7). The association of individual well-being (QLLH), with organizational integration and resources in the Argentinean-Spanish sub-sample was n.s. LpO was not correlated with QLLH in the Argentinean-Spanish sub-sample. Finally, the association of GHWB with LpO was *r* = 0.66 for the MS and 0.75 for WS, both *p* = 0.001. Transformational LpB (*r* = 0.12, *p* < 0.01) and LpO styles (*r* = 0.66, *p* < 0.001) were more strongly associated with well-being than transactional LpB (*r* = 0.09, *p* < 0.10) in MS (H6).

**FIGURE 2:**
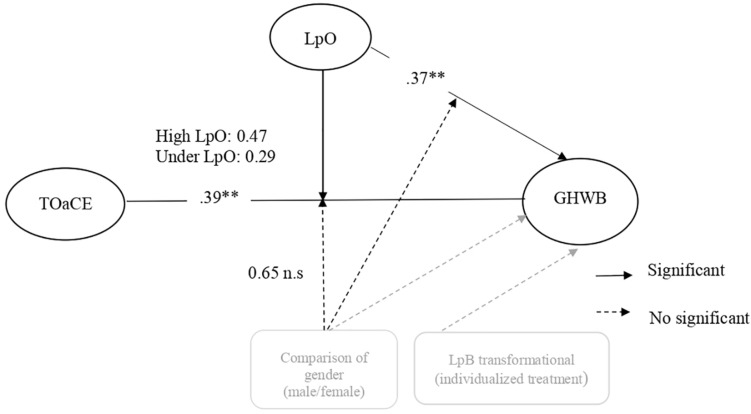
Effect of task orientation and the climate of excellence (TOaCE) in the Group Hedonic Well-Being (GHWB) moderated by the Organizational Leadership that reinforces participation and belonging (LpO) in MS. The model controls gender and leadership that reinforces participation and transformational belonging of the immediate superior in relation to the predictor variable and the result ones. All the values are standardized. **p < 0.01.

To verify H6b, moderation analyses were conducted using sex (WS and MS) and transformational leadership (individual level) (MS) as co-variables. The relation between TOaCE and GHWB β = 0.39 [0.31; 0.48], moderated by LpO *b* = 0.36 [0.28; 0.44] was positive and significant for Argentina. Gender, while it had a direct effect β = −0.10 [−0.18; −0.3] on MS, did not moderate the relation between LpB and GHWB, and it did not between TOaCE and GHWB, either (interactions are n.s., both with process and with hierarchical regression, see Table 9). Finally, controlling gender and individual approach (transformational leadership) in the MS, the analysis showed that the greater the perception of LpO favorable to participation and integration in the organization, the greater the effect of TOaCE in GHWB β = 0.47 IC [0.34; 0.59], but this effect decreases as this perception also decreases (average level β = 0.38 IC [0.29; 0.46]; and low level β = 0.29 IC [0.18; 0.39]) (Figure 2 shows results from the moderating effects). The second moderation analysis (H6b) displayed a negative association, when an organizational structure with fewer resources and less integration is perceived, the effect of the TOaCE on GHWB is greater β = 0.41 IC [0.34; 0.48] and it decreases as said perception decreases (average level β = 0.35 IC [0.25; 0.41] and lower level β = 0.31 IC [0.23; 0.39]).

**TABLE 9:** Summary of hierarchical regression analyses for moderating effects of task orientation and climate of excellence, gender and transformational style of the superior (Study 3).

Predictor variable	Step 1	Step 2	Step 1	Step 2	Step 1	Step 2
	Beta (β)	Beta (β)	Beta (β)	Beta (β)	Beta (β)	Beta (β)
TOaCE	0.36**	0.39**	0.60**	0.0.69**		
LpO	0.37**	0.37**			0.66**	0.66**
LpB	0.088*	0.089*				
Gender	−0.083*	−0.090*	−0.07^ߪ^	−0.08^ߪ^	−0.11*	−0.12*
TOaCE*LpO		0.077*				
TOaCE*Gender				0.65		
LpO*Gender						0.02
*R*2	0.758	0.763	0.484	0.89	0.441	0.449
Δ*R*2		0.05*		0.005		0.008

TOaCE, task orientation, and climate of excellence; LpO, organizational leadership that reinforces participation and belonging; LpB, leadership that reinforces participation and belonging (transformational); Gender = 1 = women, 2 = man. * *p* < 0.05; ** *p* < 0.01, ^ߪ^p < 0.10.

### Gender Differences

Regarding the third hypothesis (*N* = 900), women perceive greater group hedonic well-being in their organizations (*r* = 0.056, *p* = 0.045), specifically in Argentina [*r*_(__50__)_ = 0.39, *p* < 0.002], Mexico [*r*_(__134__)_ = 0.24, *p* < 0.002] and Brazil [*r*_(__273__)_ = 0.11, *p* < 0.04] than men. In Spain and in the military setting, correlations were n.s. Women declare a more favorable vision of group processes, specifically of TOaCE (*r* = 0.06, *p* = 0.03), although not of IPaCG (*r* = 0.03, *p* = 0.18), they perceive better LR (*r* = 0.11, *p* < 0.001), specifically for autonomy (*r* = 0.07, *p* < 0.02), a more favorable CSO or structure (*r* = 0.13, *p* < 0.001), specifically for resources (*r* = 0.18, *p* < 0.001) and better perception of LpO (*r* = 0.12, *p* < 0.001) in their organization. There are practically no gender differences in LpB (military setting) and associations are marginal. In the sub-sample in Argentina women displayed greater well-being or quality of life related to health (*r* = 0.24, *p* < 0.04) than men. Differences in stress symptoms were n.s. In Spain, men displayed greater well-being (*r* = −0.21, *p* < 0.001) than women (*n* = 0.72), but also more behavioral stress symptoms (*r* = −0.21, *p* < 0.01) (H3).

## Discussion

This study corroborated the nomological network of well-being with individual and collective-level indicators, in a sub-sample of Spanish and Argentinean workers. Reaffirming that collective affectacts as a context that influences individual well-being, the hypothesis about the relationship between climate and well-being was confirmed. 9% of the variance in well-being as quality of life linked to health was explained by emotional climate. Showing the independence of the constructs, the associations between well-being indicators were below 0.70, as postulated in the first hypothesis. Contrary to expectations and H2, values for openness to change, as well as transcendence values, did not influence how the socio-emotional climate was perceived in the military setting.

The gender hypothesis was partially verified. While in Southern Europe men report (slightly) greater hedonic group well-being (in line with the third hypothesis), in Latin America, women are the ones who perceive a more positive socio-emotional climate (and show more favorable perception of group and organizational processes). In the military sample, no significant gender differences were found. In the same vein, in this sample gender did not moderated the relationship between the climate of excellence and organizational leadership with the collective well-being. We expected that given the masculine character of the military culture, it would be the men who would benefit more from functional organizational factors to generate a good emotional climate. This was not the case, which is consistent with the fact that female cadets do not perceive a worse emotional climate. The fact that there have been recent institutional reforms to incorporate women and modernize military culture probably explain this result.

The fourth hypothesis was broadly corroborated: the less seniority at an organization, the greater the perception of a positive socio-emotional climate at the organization, and consequently group hedonic well-being. This might suggest that long-term belonging puts a benevolent view of the organization into question. The intention to remain in the organization displayed the expected profile, although the relation with well-being was only significant in Brazil and Spain. Finally, the degree of competence and prior participation with work teams or innovation is associated with group hedonic well-being, both in the worker and in the military samples.

On the other hand, the hypotheses five and six at the meso-level were confirmed, since, the greater the internal participation and communication, the task orientation and the climate of excellence, and the control of the task and the complexity of the organizational role were, the better the socio-emotional climate was. However, these variables were not associated to individual well-being as stated by H5 and 6, suggesting that these meso-level processes are very distal and do not affect individual experience, but only the organizational climate. Instead, stress was found to be negatively associated with both emotional climate and individual well-being. This result suggests that stress in the workplace plays a center role. Nevertheless, it should be noted that this variable was measured as individual stress reactions in this study, therefore, it is not possible to say whether the perception of collective stress would be associated to individual well-being.

From another angle, the perceived transformational leadership of the superior, an individual level variable, was associated in the expected sense with the socio-emotional climate. Also, as expected by H5 the relationship was weaker –although positive- with well-being, for transactional leadership. Results suggest that leadership style and behaviors have an important place in the creation of a positive organizational climate. Transformational leaders can constitute important affective events which heighten the positive feelings of their followers and seek to meet their emotional needs, reinforce satisfaction with job, creating trust and a supportive climate (Menges and Kilduff, 2015).

The perception of positive organizational leadership, as well as role complexity and the perception that the organizational culture is integrated and has resources, was associated with collective hedonic well-being, as had been postulated by H6. The macro and meso variables were strongly associated to each other and with less intensity to the individual ones, showing convergent validity. In addition, the variables of these levels -excluding climate-, were not associated to personal well-being. These result suggest that the micro and meso social influence on individual well-being transits through the socio-emotional climate, and reinforce the idea that the emotional climate of the team work acts as a context that influences personal affect and well-being- at the margin of other processes and beyond the shared experiences (Páez et al., 2013).

It was confirmed as expected by H6b that positive leadership moderates the relationship between a climate of excellence and collective well-being. The reinforcing role of task orientation and climate of excellence in relation to socio-emotional climate is more important when organizational leadership is positive and innovation-oriented, and weaker when the opposite occurs. Organizational leadership enhances the favorable role of the group process on collective well-being, thus supporting the idea that organizational leader style, and not only immediate superior leader behavior, has an important place in shaping the organizational climate and creation of a positive environment (Menges and Kilduff, 2015).

Finally, it was found that a structured and integrated organizational culture moderates the relationship between role complexity and collective well-being. A more integrated and resourceful organizational culture offset or lessen the part of role complexity in climate –even though a synergy or increase could also be expected as stated by H6b, results suggest a compensation process. The reinforcing role of complexity is more important when the culture is less integrated and weaker when the opposite occurs. The organizational strengths lessen the weight of the role complexity. As limitations of this study, it can be noted that it was correlational and that stratified random sampling was not performed.

### Study 4: Factors That Contribute to Well-Being: The Case of Work Teams in Spain

This study uses the collective well-being indicator in a sample of workers from different Spanish organizations, in a longitudinal intervention (T1 and T2). Specifically, they are workers in innovation teams. Firstly, the indicators which make up collective well-being are correlated (H1). The relationship of well-being with gender is also examined (H3); with the increased knowledge about teamwork (KPW) and the intention to stay (SO) (H4); with micro-social factors such as internal participation and safe communication (IPaCG), task orientation and climate of excellence (TOaCE), control over work (CWa), leadership that promotes participation and integration in the team (LpB) – represented in this study by transformational leadership (TL), shared leadership (SL), empowerment of the team facilitator (EMPW) – (H5). Finally, this study specifically contrasted the mediating role of leadership (TL – T1) between task orientation or climate of excellence (TOaCE) and collective well-being (GHWB) in T2 postulated by H5a.

#### Sample

The sources of information collection were the members of the work teams. The final sample was made up of 14 innovation teams (*n* = 4 y 7 members each one, *N* = 80). Participants were mostly women (*n* = 52, 63.4%; man *n* = 30, 36.6%), *M* = 35. 88 years (*SD* = 9.15), with high studies (*n* = 72, 87.8%), and a seniority in their organization less than 15 years (*n* = 65, 79.3%; from 16 to 25 years *n* = 9, 11%; more than 26 years *n* = 7, 8.5%). 53.8% were baseline workers (*n* = 43), 25. 0% intermediate commands (*n* = 28), and 11.3% belonged to the management team (*n* = 9)^19^.

#### Procedure

Different organizations were contacted through two different institutions (R + D). The organizations who showed interest signed a participation commitment and according to the methodology used. Those who were selected received a 12-h long training and sessions of monthly follow up over 5 months. These teams had to carry out a specific task (“the order”) commissioned by the organization, during a maximum period of 6 months. Information gathering was carried out before teams setting and once they had been completed.

#### Instruments

The KPW is measured with an instrument of 3 items (Martínez-Moreno et al., 2018), using a Likert-style scale of 5 anchor points (1 = never y 5 = very frequently). Cronbach’s α: 0.66 in T1. The GHWB indicator was shaped by the trust and bonding dimensions (Ayestarán et al., 2006) and satisfaction with the team (Antino et al., 2014). The first two dimensions are measured with a Likert-style scale (8 and 7 items respectively) of 5 anchor points (strongly disagree = 1 to 5 = strongly agree), (Cronbach’s α = 0.86 in T1 and 0.89 in T1 and 0.85 in T2 respectively). Satisfaction with the team is measured with Likert-style scale (2 items) of 5 anchor points (strongly disagree = 1 to 5 = strongly agree). Cronbach’s α = 0.78 (T1) and 0.80 (T2). The relation between the variables was shown to be *r* = 0.53 which allows to be unified in one single variable named GHWB. Finally, Cronbach’s Alpha of the global variable was 0.92 in T1 and 0.92 in T2. IPaCG evaluates the knowledge and management of the ICT in the job context (Martínez-Moreno et al., 2018). It is measured with a Likert-style scale (7 items) (never = 1 to 5 = very frequently). Cronbach’s α = 0.77 (T1). TOaCE is evaluated in this study through the coordination achieved in the team to develop the task and contribute to a climate of excellence. This variable was measured through 5 items (Lewis, 2003), with a response Likert-style scale of 5 anchor points (strongly disagree = 1 to 5 = strongly agree). Cronbach’s α = 0.88 in T1 and 0.89 in T2. CWa was measured through the adaptation to the work and organizational environment, an instrument of 7 items (Ayestarán et al., 2006), using a Likert-style scale of 5 anchor points (7 items). Cronbach’s α: 0.84 in T1 and 0.85 in T2. LpB is evaluated by the perceived transformational leadership in the team (Moriano et al., 2014). It is measured with a Likert-style scale (20 items) (never = 1 to 5 = very frequently). Cronbach’s α: 0.77 to 0.88 (T1) and 0.83 to 0.90 (T2). EMPW (Ayestarán et al., 2006) with a scale (7 items) of 5 anchor points (rarely or never 1 = 5 very frequently), Cronbach’s α: 0.92 (T19) and 0.93 (T2). SL in the team has been elaborated following the social media perspective (Carson et al., 2007), ergo, the team members value the leadership of each of them in the team using the scale of answer of 5 anchor points where 1 = nothing and 5 = absolutely^20^.

#### Data Analysis

Descriptive statistics, correlations and reliability are provided in Table 11. Given the effect of sex discovered above, we conducted an exploratory follow up analysis, *t*-tests, to examine whether the differences in EMPW, IPaCG and KPW were significant. To test if LpB would play a mediator role between CWa and GHWB relationship, moderated and mediation regression analyses were conducted using the *bootstrapping* method with bias corrected and accelerated confidence estimates too (Hayes, 2018).

## Results

Concerning the dimensions of GHWB, our results pointed out that trust, bonding and team satisfaction positively correlated at T1 (*r* < 0.58, *p* < 0.01) and at T2 (*r* < 0.53, *p* < 0.01). Therefore, H1 is supported by our data (see Table 10). By respect to H4, our results pointed out that SO negatively correlated with GHWB at T1, *r* = −0.23, *p* < 0.05 as expected. Our results do not supported this hypothesis, because they indicated that KPW do not significantly correlate with GHWB neither at T1, *r* = −0.14, *p* = n.s., nor T2, *r* = −0.14, *p* = n.s. Regarding H5, our data also pointed out that IPaCG do not significantly correlated with GHWB at T1, *r* = 0.19, *p* = n.s., and T2, *r* = 0.12, *p* = n.s. as expected. However, GHWB at T1 positively correlated with TOaCE at T1 (*r* = 0.75, *p* < 0.01) and at T2 (*r* = 0.34, *p* < 0.01) and GHWB at T2 is predicted by TOaCE at T1 (*r* = 0.45, *p* < 0.01) and correlated with at T2 (*r* = 0.64, *p* < 0.01) too. Thus, H6 is partially supported by our data. Table 10 also showed that GHWB at T1 and T2 positively correlated with CWa, at T1 (*r* = 0.67, *p* < 0.01 at T1; *r* = 0.42, *p* < 0.01 at T2) and is predicted by this variable at T2 (*r* = 0.39, *p* < 0.01 at T1; *r* = 0.69, *p* < 0.01 at T2). Our results underlined the positive correlation between participative leadership and GHWB, especially in the case of LpB with *r* values superior to 0.50. Specifically, TL T1 correlated with GHWB T1 *r* = 0.69, *p* < 0.01 and predicted GHWB T2 *r* = 0.50, *p* < 0.01. SL T1 correlates with GHWB T1 and predicted T2, both *r* = 0.34, *p* < 0.01. Finally in T2 TL and SL correlates with GHWB *r* = 0.64 and r = 0.46, both *p* < 0.01. EMPW T1 correlates with GHWB T1 *r* = 0.50 and predicted GHWB T2, *r* = 0.42, both *p* < 0.01. In T2 EMPW correlates with GHWB T2 *r* = 0.58 *p* < 0.01. Therefore, results support H5.

**TABLE 10:** Association between the variables that make up the group hedonic Well-Being at the team level (Study 4).

	*N*	Mean	*SD*	1	2	3	4	5	6
Trust T1	79	4, 00	0.64	–					
Trust T2	39	3, 97	0.59	0.65**	–				
Bonding T1	76	4, 19	0.66	0.58**	0.47**	–			
Bonding T2	75	4, 34	0.62	0.35**	0.67**	0.38**	–		
Team satisfaction T1	76	4, 46	0.76	0.65**	0.41**	0.59**	0.27*	–	
Team satisfaction T2	75	4, 43	0.68	0.38**	0.55**	0.26**	0.53**	0.43**	–

* *p* < 0.05; ** *p* < 0.01.

**FIGURE 3:**
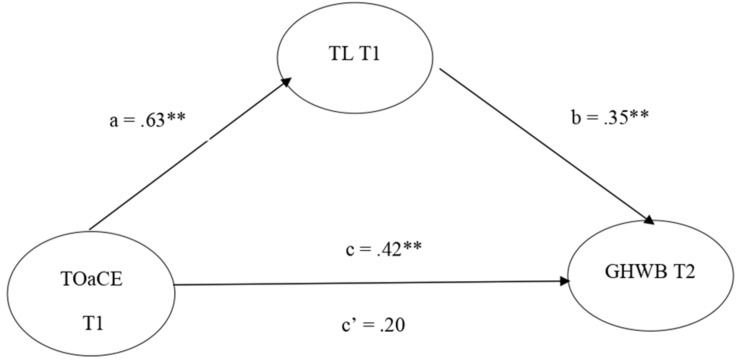
Effect to task orientation and climate of excellence on group hedonic Well-Being (GHWB T2), mediated by transformational team leadership (TL T1) [β = 22, *SE* = 19, 95%] CI 95% [0.04; 0.42] **p < 0.01.

In accordance with the mediational facet of H5a, that LpB at T1 would play a mediating role between TOaCE at T1 and GHWB at T2, results indicated that TOaCE at T1 was positively related to GHWB at T2 when controlling for IPaCG [β = 0.42, *t*_(__66__)_ = 3.85, *p* = 0.01] and they also confirmed mediating role of LpB at T1 in this relationship because the direct effect of TOaCE on GHWB became non-significant when LpB mediator is included in the equation [β = 0.20, *t*_(__65__)_ = 1.47, *p* = 0.15]. The overall model was significant: *R*^2^ = 0.29, *F*(3,65) = 7.94, *p* < 0.001 (see Figure 3).

### Gender Differences

By respect to H3, as Table 11 showed, gender (meaning that men score higher) correlates negatively but not significantly with GHWB T1 and T2. Sex also correlated with IPaCG (*r* = −0.24, *p* < 0.05), CPE (*r* = −0.26, *p* < 0.05), and empowerment at T1 (*r* = −0.38, *p* < 0.01) and at T2 (*r* = −0.28, *p* < 0.05). Additional *t*-tests conducted showed significant differences between women and men in PyCG, *t*_(__77__)_ = −2.35; *p* = 0.02, in KPW, *t*_(__45__)_ = −2.05; *p* = 0.05, and in EMPW at T1, *t*_(__75__)_ = −3.48; *p* = 0.01 and at T2, *t*_(__72__)_ = −2.52; *p* = 0.01. Our data indicated that women have less experience in working in teams (*M* = 3.28, *SD* = 1.23) and using ICTs (women, *M* = 2.75, *SD* = 0.75) than men (experience in teamwork, *M* = 3.11, *SD* = 0.66; PyCG, *M* = 3.11, *SD* = 0.66). They also perceive less empowerment at T1 (*M* = 3.71, *SD* = 0.72) and T2 (*M* = 3.81, *SD* = 0.73) than men (EMPW T1, *M* = 4.28, *SD* = 0.58; EMPW T2, *M* = 4.2 *SD* = 0.62).

**TABLE 11:** Association between individual, micro-social, and Well-Being level variables (Study 4).

	*N*	Mean	*SD*	1	2	3	4	5	6	7	8	9	10	11	12	13	14	15	16
(1) Gender	82	–	–	–															
(2) SO	82	–	–	0.01	–														
(3) KPW	79	3.51	1.23	−0.26*	–0.06	–													
(4) IPaCG	79	2.87	0.73	−0.24*	–0.15	0.27*	–												
(5) GHWB T1	79	4.11	0.59	–0.08	−0.23*	–0.14	0.19	–											
(6) GHWB T2	75	4.29	0.55	–0.22	0.04	–0.14	0.12	0.40**	–										
(7) TOaCE T1	77	4.10	0.63	–0.11	−0.22*	–0.05	0.19	0.75**	0.45**	–									
(8) TOaCE T2	75	4.26	0.70	–0.20	–0.05	0.08	0.21	0.34**	0.64**	0.49**	–								
(9) CWa T1	77	3.85	0.72	–0.08	–0.21	–0.01	0.03	0.67**	0.42**	0.69**	0.33**	–							
(10) CWa T2	75	3.96	0.77	–0.21	–0.05	–0.02	0.26*	0.39**	0.69**	0.51**	0.67**	0.54**	–						
(11) LpB transformational T1	77	3.83	0.63	–0.18	–0.21	–0.02	0.23*	0.69**	0.50**	0.67**	0.52**	0.62**	0.56**	–					
(12) LpB Transformational T2	74	3.97	0.65	–0.17	–0.15	–0.05	0.26*	0.57**	0.64**	49**	0.59**	0.42**	0.63**	0.72**	–				
(13) SL T1	78	3.89	0.75	–0.13	–0.12	0.05	0.12	0.34**	0.34**	0.51**	0.36**	0.50**	0.39**	0.38**	0.30*	–			
(14) SL T2	75	3.85	0.74	–0.03	0.04	–0.04	0.20	0.32**	0.46**	0.36**	0.43**	0.23	0.49**	0.31**	0.54**	0.36**	–		
(15) EMPW T1	77	3.90	0.73	−0.38**	–0.17	0.10	0.27*	0.50**	0.37**	0.57**	0.43**	0.43**	0.54**	0.66**	0.61**	0.36**	0.39**	–	
(16) EMPW T2	74	3.97	0.71	−0.28*	–0.06	0.05	0.31**	0.42**	0.58**	0.43**	0.52**	0.42**	0.65**	0.64**	0.80**	0.26*	0.41**	0.66**	–

Gender (1 = women, 2 = man); SO, seniority in the organization; KPW, degree of knowledge and previous participation in work teams; IPaCG, internal participation and communication in the group; GHWB, group hedonic Well-Being; TOaCE, task orientation, and climate of excellence; CWa, control over work, role autonomy; LpB, leadership that reinforces participation and belonging (transformational); SL, shared leadership; EMPW, empowerment. * *p* < 0.05; ** *p* < 0.01.

## Discussion

This last study has focused on analyzing which variables at the micro-social level are related to positive socio-emotional climate. Results support H1 because GHWB indicators correlates strongly, but lower than 0.70. With respect to H3, collective well-being was higher in men, but not significantly so. However, women in general showed lower scores than men on factors favorable to well-being. We believe that this is explained because, in this study, women have stated that they have less experience both in the degree of knowledge and management of ICTs and in previous experience in teamwork. Previous studies have shown that the perception of gender equality at work enhances the well-being of workers, especially women’s (Chawla and Sharma, 2019), therefore human resources policies, which facilitate training in basic knowledge for the development of their work and that allow greater empowerment could contribute to a greater well-being of this group. H4 was supported by results, because seniority was negatively related to collective well-being. However, knowledge and experience in team work did not predict emotional climate. In this sense, this part of H4 was unsupported.

Globally H5 and 6 were confirmed: TOaCE and CWa predicted GHWB, showing how implicit coordination and adaptation to the team’s environment are group processes that determine not only the functioning of the team but also the socio-emotional climate that is created in it, including a climate of trust and greater satisfaction in its members. Likewise, as Müller and Antoni (2020) point out, this study also shows that the degree of knowledge and management of ICTs is important for establishing greater implicit coordination in the team, which in turn will allow to probably develop a favorable socio-emotional climate. The team’s ability to adapt to its environment and to the workload imposed by the task involves the division and distribution of tasks and support among team members. Feeling the support of team members when you are overwhelmed by the task relieves tension and stress (Seibert et al., 2011; Chawla and Sharma, 2019). Therefore, adaptation to the work environment and workload, in addition to being a clear antecedent of team performance (DeChurch and Mesmer-Magnus, 2010; de Wit et al., 2012; McEwan et al., 2017), also favors a favorable socio-emotional climate (Menges and Kilduff, 2015).

Results confirm the mediating role of transformational leadership between TOaCE and collective well-being, stated by H5a. This study has revealed the role of participatory leadership as a key factor for the development of a favorable socio-emotional climate in work teams. How the people coordinating the team exercises their leadership, their ability to empower and delegate responsibilities to team members contributes to the well-being of the team. When the transformational leader is able to empower members and delegate responsibilities, shared leadership can emerge (Davidson, 2020; Martínez-Moreno et al., 2018). Interestingly, the empowerment is perceived to a lesser degree in this sample by women. This study has some important limitations. On the one hand, it presents a reduced sample, so future studies should replicate it with a larger sample that allows for more complex analyzes that determine the associations between variables described here. On the other hand, the data collection has been carried out only through self-reports, so it would be interesting to deepen the study of socio-emotional climate through other measurement instruments, such as interviews or focus groups.

## Summary and Conclusion

This investigation examined the factors of well-being related to belonging and social integration at *individual, micro* and *meso* social levels. This issue was addressed in organizations from 6 countries, specifically in the labor and military fields. The main results of four studies can be concluded as follows (see Table 12):

**TABLE 12:** Main results of the studies and global effect size of two or more studies using the same predictive and well-being construct-at similar individual or collective level.

	Study 1 Individual Cross sectional Explained variable	Study 3^a^ Individual Cross sectional Explained variable	Study 3 Collective Cross sectional Explained variable	Study 4 Collective Longitudinal Explained variable	Conclusion
Predictive variables and hypothesis	EPWB *N* = 1.300	QLLH *N* = 350	GHWB *N* = 1078	GHWB *N* = 80	Hypothesis were
**H1:** Confirming the nomological network of well-being, the indicators of individual level and collective level will be associated with each other. Showing the independence of the constructs, the associations between well-being indicators will be lesser than 0.70.	Supported average correlation between all pairs well-being indicators $\bar{r}$_(1__7__)_^b^ = 0.42 range −0.35 (negative affect) to 0.58	*r* = 0.29 with collective Well-Being or climate		Supported average correlation between GHWB indicators $\bar{r}$_(2__)_ = 0.55 Rang 0.26 to 0.65	Supported for individual (in one study) and collective well-being weighted $\bar{r}$_(__30__)_^c^ = 0.34
**H2**: Individual-level predictors values of openness to change and self-transcendence will be associated with well-being. PVQ scale study 1 and 3	*r* = 0.25 and *r* = 0.23	–	*r* = 0.02 WS and *r* = 0.03 MS n.s.	–	Supported for individual well-being in one study Individual variables are unrelated to collective well-being
**H2** EC as a particular trait, will be associated with positive and negative affectivity.	*r* = 0.14 EPWB *r* = 0.21 positive AHWB *r* = 0.17 negative AHWB	–	–	–	Supported for individual well-being in one study
**H3:** Minor differences in well-being will be found and these will favor males.	Chile *r*_(__333__)_ = 0.03, *p* = 0.27; Spain *r*_(__283__)_ = −0.091, *p* = 0.06; Uruguay *r*_(__675__)_ = 0.02, *p* = 0.33	–	Women report higher collective well-being *r* = 0.056, *p* = 0.045, in all but two samples	*r* = −0.08 n.s.	Absence or Minor differences in favor of males for individual well-being Unsupported Minor differences in favor of females for collective level weighted $\bar{r}$_(__1__878__)_ = 0.046
**H4:** The less seniority and the greater knowledge and participation in work team, commitment and job satisfaction (agreement with organizational methods and intention to stay), the greater the well-being.	SO = 0.01 n.s. AM *r* = 0.29 IS *r* = 0.13	–	SO *r* = −0.16 IS = n.s. KPW *r* = 0.13	SO *r* = −0.23 KPW *r* = −0.14 n.s.	Supported for collective well-being *for SO*$\bar{r}$ (1 138) = −0.16 Supported in one of two studies for IS Supported for collective well-being KPW weighted $\bar{r}$_(1__138__)_ = 0.11
**H5**: The micro-social predictive factors (control over work and leadership that reinforce participation and belongingness) will be associated with both individual and collective well-being. (Istas 2 CWa study LpB Istas 4 study 1	*r* = 0.33 *r* = 0.28	–			Supported for individual well-being
**H5**: Stress will be negatively associated to well-being. Istas1 EPs Study 1 and BSCs Setterlind scale Study 3	*r* = −0.28	*r* = 0.50 BSCs (positive scores means low stress)	*r* = 0.19	–	Supported for individual well-being weighted $\bar{r}$_(__1__650__)_ = −0.33 and collective level
**H5:** Transformational leadership, and shared and quality leadership, will be associated with well-being. LpB Transformational leadership style Nader’s scale Study 3 and Moriano’s scale Study 4, shared leadership scale SL and quality leadership EMPW scale in Study 4			*r* = 0.15	*r* = 0.50 *r* = 0.34 *r* = 0.42 $\bar{r}$_(3)_ = 0.42	Supported for collective well-being weighted $\bar{r}$_(__1388__)_ = 0.17
**H5:** At the team level, transformational leadership will mediate between group coordination or autonomy and collective well-being.				Mediational analysis confirm that transformational leadership mediates between group coordination/autonomy and emotional climate	Supported for collective well-being
**H6**: Predictive factors at the mesosocial level like orientation to work and participation and communication will be associated with well-being. TOaCE and IPaCG FINO scale in study 3, knowledge and management of the ICT in the job context and coordination achieved in the team Lewin’s scale in study 4	–	n.s. correlations	*r* = 0.74 WS and 0.63 MS *r* = 0.68 *r* = 0.76 WS and 0.69 MS *r* = 0.74	*r* = 0.45 *r* = 0.12	Supported for collective well-being in two studies but no association with individual well-being
	**Study 1** **Individual** **Cross sectional** **Explained variable**	**Study 3^a^** **Individual** **Cross sectional** **Explained variable**	**Study 3** **Collective** **Cross sectional** **Explained** **variable**	**Study 4** **Collective** **Longitudinal** **Explained** **variable**	**Conclusion**
**H6:** Role autonomy will be associated to well-being LR organizational (FINO) Study 3 and adaptation to the work and organizational Ayestaran’s scale Study 4		n.s. correlations	*r* = 0.71 WS and 0.69 MS	*r* = 0.39	Supported for collective well-being
**H6**: LeR having to express emotions, especially negative ones, will be negatively associated with individual well-being. Expression of negative, neutral and positive emotions	*r* = -0.23, *r* = -0.10 *r* = 0.05 n.s.	–	–	–	Supported for expression of negative and neutral emotions for individual well-being in one study
**H6:** Emotional dissonance LeR will be negatively associated to well-being	*r* = −0.17	–	–	–	Supported for individual welllbeing in one study
**H6:** CSO Transformational vs. transactional organizational culture ODQ in Study 1 Transformational culture will be positively and more strongly associated with well-being than transactional culture High integration and resources in the organization will be positively related to well-being OS FINO’scale in Study 3	*r* = 0.21 *r* = −0.08	– n.s. correlations	– *r* = 0.73 WS and 0.64 MS	–	Supported for individual well-being in one study and only for collective well-being collective level in another
**H6:** Positive organizational leadership will be associated to well-being LpO FINO’s scale Study 3		n.s. correlations	Positive organizational leadership *r* = 0.78 WS and0.66 MS		Supported for collective not individual well-being
**H6:** Transformational leadership will be associated with well-being and more strongly than transactional leadership. LpO Nader scale Study 3	–		Transformational *r* = 0.12 MS Transactional *r* = 0.09 MS	-	Supported for collective well-being but effect size are not so different
**H6a:** Psychosocial factors will mediate between organizational culture and individual well-being.	Mediational analysis support that stress, control of work, and leadership mediates between transformational culture and well-being	–	–	–	Supported in one study and MS
**H6b:** The positive relationship between variables and group climate will be moderated by an organizational leadership favorable to participation. Gender (being male) moderates strength of association between variables and group climate			Moderation analysis confirms that the association between orientation to work and emotional climate was strong when level organizational leadership was high. Moderation analysis did not found a gender effect		Supported collective level in one study Unsupported
**H6b:** The positive relationship between variables and group climate will be moderated by an inclusive organizational structure with resources.			Moderation analysis disconfirms that the association between orientation to work and emotional climate was strong when level of organizational structure and resources was high.		Unsupported: results suggest compensation effect

*^*a*^Study 2 was not included because educators data was used in the first study and include them would mean using their data twice. ^*b*^This are the number of paired correlation that were aggregated. ^*c*^This are the total number of subjects in which weighted correlation was calculated.*

(1) Nomological well-being networks were explored, using, at individual level, quality of life linked to health, hedonic affective and cognitive well-being, as well as psychological well-being, verifying that they are congruently associated with each other. So do trust, bonding, and satisfaction with participation at the team level, and the socio-emotional climate with cohesion at the organizational level. This allows us to conclude in concordance with H1 that the nomological network is a representation of the concepts or constructs of interest and the interrelations among them. Consistent with the meta-analysis of Parker et al. (2003), where the warm and cooperative psychological climate in work teams is associated with eudemonic well-being, the socio-emotional climate in this research was associated with psychological well-being acting as a personal context.
(2) Consistent with H2 and previous studies (Soroa et al., 2015), emotional creativity is associated with well-being, although in an ambivalent way, since it is also associated with negative affectivity and greater reactivity to stress. High attention and cognitive processing of intense feelings, difficult to categorize, label and express novel and complex emotions, typical of people with high EC, has negative effects on coping and well-being (Averill, 2009). Also congruent with previous studies, those who value self-direction, stimulation, gratification, as well as justice and well-being for all, report better psychological well-being. Openness to change and universalism are cognitive representations of hedonist, self-actualization and expansion of self or growth needs. Motivation to pursue these values helps to satisfies hedonic, competence and relatedness need, fueling benevolent and prosocial perceptions, attitudes and behaviors increase well-being (Schwartz and Sortheix, 2018). However, personal values were not correlated with collective level well-being. These results suggest that the socio-emotional climate is less influenced by personal variables and more by micro and meso-social variables. It could also be suggested that it is through personal well-being that values indirectly influence emotional climate.
(3) Regarding gender, consistent with previous studies and H3, the results show that men have the highest positive affective well-being in education and social intervention (Study 1). However, women report greater collective well-being, specifically, in the workplace in Latin America. This result would be consistent with the meta-analysis of Tifferet (2020), where it indicates that women have a more favorable perception of the environment. Women would give and receive more social support than men by establishing broader social networks online. However, this result did not occur in the military sphere. Carreira (2017) analyzes how, from the standpoint of culture and structure, hegemonic definitions of the military are combined with ideology and male hegemonic culture. In this way, the military has long been a source of normative conceptions of gender and a space for the construction of male identity. Halberstam (2008) invites us to think about masculinity and femininity beyond the bodies of men and women, especially to create awareness in some institutions that have built their organizational culture largely on the basis of masculine and masculine images that dominate organizational processes segregating women and the feminine. In Spain, although men report greater well-being than women, they also report greater behavioral symptoms of stress (Study 3). In this country, women would have less experience in teams of excellence and in the use of ICTs, as well as less satisfaction with the leadership style of those who facilitate them (Study 4).
(4) Increased commitment to the organization, this means agreement with methods, in one study, and intention to remain, in one study but not in another, were associated with individual well-being. Knowledge and experience in teamwork were related to collective well-being in them with a weak effect size, confirming that teamwork enhance group climate (De Jong et al., 2016). Seniority was unrelated with individual well-being, but was negatively related with collective well-being as expected.
(5) Reaffirming the importance of work psychosocial factors (Moncada et al., 2014), and congruently with H5, the greater control of work and leadership that reinforces participation and belongingness, the higher the individual well-being levels in study 1 (and also in study 2 teachers sample). Effect sizes were strong explaining around 9% of variance. These results highlight that a high level of control at work, the autonomy, complexity and challenge of the work role constitute a basis for active work, learning and greater performance, while reinforcing individual and collective well-being. Also reaffirms that a positive leadership and social support in the team work are important for well-being (Moncada et al., 2014). Furthermore, stress was negatively associated with both emotional climate in two studies (weak effect size) and well-being in one study (strong effect size). Qualitative and quantitative work overload erodes individual well-being an affect negatively emotional climate. In the same sense, in concordance with H5 the safer the participation and communication and the greater the orientation to the task, the better the emotional climate. However, these variables were not associated with individual well-being, suggesting that they are very distal processes that do not affect individual experience, but only organizational climate (see Study 3 discussion).
(6) The relevance of leadership that facilitates participation and integration stated by H6 was widely corroborated, with a medium effect size for collective well-being in study 3 and 4, thus reaffirming that functional, inspiring and motivating forms of leadership, such as transformational leadership styles, which are based on empowering and sharing with the members, reinforce socio-emotional climate (Arnold, 2017). This result is in line with meta-analyzes that found that supervisors’ social support is associated with well-being (Kossek et al., 2011; Eby et al., 2013; Mathieu et al., 2019). Moreover, positive leadership at team level (Study 4) and organizational level (Study 3) play a mediational role between functional group and organizational factors and collective well-being, adding evidence to the pivotal role that leadership has in the dynamics of the organizational climate (see discussion Study 3 and 4).
(7) Supporting H6, a more complex labor role at the organizational level predicts collective well-being as did the micro-level job control related to H5, which emphasizes complexity, autonomy, and challenge as part of the occupational role. This control and autonomy of the work role would encompass influence, development possibilities and the meaning of work (Moncada et al., 2014). The demands of emotional labor are negatively associated to well-being, with a medium effect size, confirming that having to express neutral and negative emotions, suppressing and pretending emotions to fulfill the expectations of the work role, have a cost for the well-being (Hülsherger and Schewe, 2011). However as Study 3 showed, emotional dissonance is less frequent that the expression of negative emotions in teachers experience, suggesting that the importance of the former has been overstressed.
(8) The perception of an inclusive and participatory organizational culture is directly associated with and predicts personal well-being, with a medium effect size. They also predict it indirectly through less stress, greater job control, and more support from peers and leadership that reinforces participation and belonging, controlling for individual characteristics such as emotional creativity and motivational values – in agreement with H6 and H6a. In addition, it confirms that the transformational culture is a framework that increases social belonging through the support of peers and supervisors, as well as buffer stress and facilitating the autonomy of its members. Moreover, is a good example of explanation by articulation of the level of analysis (Doise and Valentim, 2015).
(9) The perception of positive organizational leadership in the organization and of an integrated and resourceful organizational culture was associated with a better socio-emotional climate. Organizational leadership facilitates the creation of a climate of trust and positive affectivity in the workplace, because a good quality relationship will allow subordinates to have more autonomy and freedom of decision, as well as to feel guided and motivated to work. Support from the general supervisor also reinforces performance and well-being (Hammond et al., 2011). Effect sizes for these variables are strong than previous ones, probably because of common method variance, but also because collective level measures reflect perceptions of general attitudes and behaviors, and are more stable macro-psychological indicators (Páez et al., 2013).
(10) Among other limitations, it can be noted that the samples were of convenience, and that three studies are correlational, so the results should be viewed with caution. Although the last study is longitudinal, it cannot be guaranteed that the associations between variables are causally related. Another limitation has to do with the gender variable. In this research, it was measured as a set of subjects belonging to the same sex, although some of the studies give a third option to the binary. It is necessary to collect the recommendations of the current studies (Rich-Edwards et al., 2018; Vergoossen et al., 2020) for future research.
(11) As future lines of research, we point out that the articulation between meso and micro factors in longitudinal studies are important, as well as integrating behavioral and hetero-evaluated indicators of the constructs in the studies.

## Data Availability Statement

The raw data supporting the conclusions of this article will be made available by the authors, without undue reservation, to any qualified researcher.

## Ethics Statement

Human studies were part of larger projects in each of the participating countries. These projects were reviewed and approved by the Ethics Committee of the Universidad de la Rep blica Oriental del Uruguay (UDELAR), by the participating Chilean Universities, the UFB in Brazil, the Burgos University (UBU) in Spain and the Evaluation Commission of the Argentine Ministry of Defence, which including the UNDEF. Patients/participants provided written informed consent to participate in this study.

## Author Contributions

SdC designed the study. SdC, EM-M, DM, and GE conducted the analyzes. SdC, GE, DM, AT, and DH contributed to the Studies 1 and 2. SdC, AT, EM, SG-M, VD, AA, ST, and SP contributed to Study 3. EM-M conducted the Study 4E and VD collaborated in it. All the authors contributed to the article and approved the submitted version.

## Conflict of Interest

The authors declare that the research was conducted in the absence of any commercial or financial relationships that could be construed as a potential conflict of interest.

## Footnotes

1 See acronyms in document: on line resources 1.

2 See level of analysis, predicted and predicted variables, study of the article to which it is applied, measurement instrument and example of items in document: on line resources 2.

3 In this review of emotional climate, studies were included on group affect or moods, because as a construct, this includes emotions. Moreover, group moods often represent traces of past collective emotions (Menges and Kilduff, 2015) and act as the context wherein emotions may arise (Páez et al., 2013). A predominant pleasant mood is a defining element of a positive emotional climate.

4 In Study 4, implicit coordination is analyzed, since *ad hoc* teams must carry out a specific task in a period of 6 months, going from a transition phase (training phase) to an action phase (operation phase).

5 See sociodemographic data of the participating sample by country from Study 1 in: on line resources 3.

6 See Relationship between psychological well-being and other forms of well-being by nation in table one: on line resources 4.

7 See online resources 4.

8 Only significant correlations are shown, all other scores are available on request.

9 The school in Talca (Group 1) shows a School Vulnerability Index (SVI) (Herrera et al., 2017; https://www.junaeb.cl/ive; Turra et al., 2015; López et al., 2017) of 85%.

10 JUNAEB (2020) defines vulnerability as “a dynamic condition resulting from the interaction of multiple individual and contextual risk and protection factors that are present in a person’s life cycle.” These manifest themselves “in behaviors or events of greater or lesser risk (biological, psychological, social, economic, cultural and/or environmental)” that can be measured for intervention, taking into account the development and well-being of individuals, families and communities. JUNAEB uses the National System of Allocation with Equity (SINAE) to evaluate school vulnerability (conditions of poverty and school risk) in infant education up to university level. The IVE -an indicator used by SINAE in the framework of JANAEB’s School Feeding Programmes (PAE) – measures the approximate risk of dropping out of school, through the socio-economic evaluation of its students, operating as a criterion for the allocation of PAE resources. The IVE reflects the percentage of school students with priority to receive this aid. Students in the first category or priority are those in extreme poverty, or who are destitute in urban areas or poor in rural areas, or who belong to SENAME (national service for minors), according to the social protection sheet (FPS). Students in the second category or priority are those who according to the FPS are in a situation of poverty and have a high probability of dropping out of the school system. Those in the third category or priority are those who are in poverty but do not qualify for the first two categories. The IVE measures the percentage of students in the school who are in one of the three categories. This indicator, which ranges from 0 to 100, is defined by the addition of students in these categories over the total of students. For instance an IVE of 83 means that eighty-tree students over one hundred are in risk of poverty (Holtz Guerrero, 2020).

11 https://www.ceip.edu.uy/tiempo-completo-modelo-pedagogico-fundamentacion/

12 See Study 1 and Sociodemographic data of the participating sample by country in: on line resources 5.

13 Other results were that 68% showed risk scores in the reward dimension (measuring the balance between the effort invested in completing the tasks and the reward obtained for them) and 57% in the dual presence dimension (measuring the concern with fulfilling domestic and work tasks).

14 See scales and instruments at: on line resources 2.

15 Given that the N is small and therefore the statistical power is lower, only the significant values are highlighted in the text.

16 16See general and specific socio-demographic data by country participating in: on line resources 6.

17 If not mentioned, they are instruments used on both samples (MS and WS).

18 See scales and instruments at: on line resources 2.

19 See sociodemographic data of the participating sample by country in: on line resources 7.

20 See scales and instruments at: on line resources 2.
