# Supplementary material for: Belonging and Social Integration as Factors of Well-Being in Latin America and Latin Europe Organizations
Source: Front Psychol. 2020 Dec 9;11:604412. doi: 10.3389/fpsyg.2020.604412 (PMC7756150; doi:10.3389/fpsyg.2020.604412)
Supplement: Supplementary file 1 [file Table_1.pdf]

Belonging and Social Integration as Factors of Well-Being in Latin America and Latin Europe Organizations

Frontiers of Psychology

Silvia da Costa[1*], Edurne Martínez-Moreno[1], Virginia Díaz[1], Daniel Hermosilla[1], Alberto Amutio[2], Sonia Padoan[1], Doris Méndez[4], Gabriela Etchebehere[5], Alejandro Torres[6], Saioa Telletxea[3] and Silvia García Mazzieri[7]

[1]Department of Social Psychology, Faculty of Psychology, University of the Basque Country, San Sebastian, Spain

[2]Departament of Social Psychology, Faculty of Labour Relations and Social Work, University of the Basque Country, Leioa, Spain

[3]Departament of Social Psychology, Faculty of Labour Relations and Social Work, University of the Basque Country, Vitoria, Spain

[4]Departament of Psychology, Faculty of Psychology, University of Talca, Talca, Chile

[5]Institute of Psychology, Education and Human Development, Faculty of Psychology, University of the Oriental Republic of Uruguay, Montevideo, Uruguay

[6]Argentine National Defense University, Argentina

[7]Departament of Psychology, Regional Faculty of the National Technological University, Trenque Lauquen, Argentina

Corresponding author: Silvia da Costa e-mail: silviacristina.dacosta@ehu.eus

Online Resource 1. List of abbreviations or acronyms used in the paper or in the online resources

ACBC	Autonomous Community of the Basque Country
AHWB	Affective hedonic view of subjective Well-Being
AM	Agreement with the methodology in the workplace
BSCs	Behavioral, somatic and cognitive reactions to stress
CSO	Culture and organizational structure that reinforces participation and integration
CWa	Control over work, role autonomy
DV	Dependent variable
EC	Emotional Creativity
EMPW	Empowerment
EPs	Excess of psychological demands at work or work stress
EPWB	Eudaimonic vision or psychological Well-Being and personal optimal development
EWB	Eudaimonico Well-Being
GHWB	Group hedonic Well-Being
ICT	Information and communication technology
IPaCG	Internal participation and communication in the group
IS	Intention to stay
ILO	International Labor Organization
KPW	Degree of knowledge and previous participation in work teams
LR	Labor Role
LpB	Leadership that reinforces participation and belonging
LpO	Organizational leadership that reinforces participation and belonging
LeR	Emotional Labor role
MS	Military sample
MV	Motivational values
OC	Organisational culture
OS	Organisational structure
OVC	Openness to change values
QLLH	Quality of Life linked to health
SL	Shared leadership
SO	Seniority in the organization
PWB	Psychological or Eudaimonic Well-Being
SHWB	Subjective or Hedonic Well-Being
SWB	Social Well-Being
SWL	Cognitive hedonic view of subjective Well-Being or satisfaction with life
TL	Transformational Leadership
TOaCE	Task orientation, and climate of excellence
TV	Transcendences values
TVB	Values of transcendence Benevolence
TVU	Values of transcendence Universalism
WS	Workers sample
