# Supplementary material for: Belonging and Social Integration as Factors of Well-Being in Latin America and Latin Europe Organizations
Source: Front Psychol. 2020 Dec 9;11:604412. doi: 10.3389/fpsyg.2020.604412 (PMC7756150; doi:10.3389/fpsyg.2020.604412)
Supplement: Supplementary file 2 [file Table_2.pdf]

Belonging and Social Integration as Factors of Well-Being in Latin America and Latin Europe Organizations

Frontiers of Psychology

Silvia da Costa[1*], Edurne Martínez-Moreno[1], Virginia Díaz[1], Daniel Hermosilla[1], Alberto Amutio[2], Sonia Padoan[1], Doris Méndez[4], Gabriela Etchebehere[5], Alejandro Torres[6], Saioa Telletxea[3] and Silvia García Mazzieri[7]

[1]Department of Social Psychology, Faculty of Psychology, University of the Basque Country, San Sebastian, Spain

[2]Departament of Social Psychology, Faculty of Labour Relations and Social Work, University of the Basque Country, Leioa, Spain

[3]Departament of Social Psychology, Faculty of Labour Relations and Social Work, University of the Basque Country, Vitoria, Spain

[4]Departament of Psychology, Faculty of Psychology, University of Talca, Talca, Chile

[5]Institute of Psychology, Education and Human Development, Faculty of Psychology, University of the Oriental Republic of Uruguay, Montevideo, Uruguay

[6]Argentine National Defense University, Argentina

[7]Departament of Psychology, Regional Faculty of the National Technological University, Trenque Lauquen, Argentina

Corresponding author: Silvia da Costa e-mail: silviacristina.dacosta@ehu.eus

On line resources 2. Level of analysis, predicted and predicted variables, study of the article to which it is applied, measurement instrument and example of items.

Well-being as a dependent or explained variable

Level	VD or predicted variable	Study in which appears	Measuring instrument	Sample item
Individual	BSCs	S1 (WS)<br>S2 (WS)<br>S3 (WS)	Setterlind, (2001, in Moncada et al., 2014)	During the last 4 weeks...<br>"I couldn't sleep well" (Behavioral)<br>"Have you felt any pain or tightness in your chest? (Somatic)<br>"Have you had trouble making decisions?" (Cognitive)
	QLLH		SF36 (Ware & Sherbourne, 1992)	In general, you would say that your health is: Excellent Very good Regular Bad<br>"Have you felt calm and quiet? (mental health)";<br>"Have you felt exhausted? (Vitality)
	AHWB		DESm (Fredrickson, 2009)	What is the most love, closeness, or trust you felt? (Love); What is the most stressed, nervous, or overwhelmed you felt? (Stress)
	SWL		SWL (Diener, 1996)	How satisfactory do you consider your life in the following aspects: family, money and income, work, your person, life in general.
	EPWB		PHI (Hervás & Vásquez, 2013)	"I am very satisfied with my life" (HWB)<br>"My life is full of learning experiences and challenges that make me grow" (EWB)<br>"I think that I live in a society that lets me fully realize my potential" (SWB)
Microsocial	GHWB	S3 (WS and MS)	FINO (da Costa et al., 2016)	"The social emotional climate among this organization's members is characterized by... Being positive (good humor, enthusiasm, understanding...)" (Socio-emotional climate).<br>"In the working groups of this organization: collaboration among workers prevails" (Group Cohesion)
		S4 (WS)	Ayestarán, Aritzeta and Gavilanes (2006).	The team helps us to know better our possibilities of improvement" (Confidence)<br>"In our team there has been a lot of sensitivity to the suffering of people" (Bonding)
			Gladstein (1984).	"I am happy with the way my teammates and I have worked" (Satisfaction with the team)

Well-being's predictors variable

Level	Predictor	Study in which appears	Measuring instrument	Sample item
Individual	EC	S1 (WS)<br>S2 (WS)	ECI (Soroa et al., 2015)	I think about my emotional reactions and try to understand them" (Preparedness). "I have felt emotions that other people probably have never experienced)" (Novelty). "The way I express and experience my emotions helps me in my relationships with others" (Effectiveness and Authenticity)
	MV	SI (WS)<br>S2 (WS)<br>S3 (MS)	PVQ 21 (Schwartz, 2001)	Thinking up new ideas and being creative is important to him. He likes to do things in his own original way (Openness to change, self-direction). "He thinks it is important that every person in the world be treated equally. He wants justice for everybody; even for people he does not know (Self-transcendence, Universalism).<br>It is important to him to listen to people who are different from him. Even when he disagrees with them, he still wants to understand them (Self transcendence, benevolence)
Socio-demographic variables	Gender<br>SO	S1, S2, S3, S4	da Costa, 2018; Martínez-Moreno and others (2018)	Women, man, other<br>Year of incorporation into your current organization (E1, E2, MT)<br>Year of incorporation to the job you are currently doing in your organization (E3, MT)<br>Years of work in the organization (E4, MT)
	KPW	S3 (WS, MS)	FINO (da Costa and others 2016)	I have received training on: a) work teams, b) innovation teams; I have been part of a team: a) work, b) innovation
		S4 (WS)	Martínez-Moreno and others (2018)	Have you ever worked as a team in your current organization?<br><br>I have received training on: a) work teams, b) innovation teams; I have been part of a team: a) work, b) innovation<br>Have you ever worked as member of a team in your current organization?
	AM	S1 (WS)<br>S2 (WS)	da Costa (2018)	Do you agree with the working method - methodology - used in your workplace/organization?
	IS		da Costa (2018)	I intend to remain/not intend to remain in the current job indefinitely or in the long term (E1, E2)<br>Type of contractual relationship (E3)
Microsocial	IPaCG	S3 (WS and MS)	FINO (da Costa and others, 2016)	In the working groups of this organization: decisions are taken in a consensual manner (Internal communication and secure participation)
		S4 (WS)	Martínez-Moreno and others (2018)	In general, how often have you used the following information and communication technologies in your work? (Degree of knowledge and management of ICTs in the team)

	TOaCE	S3 (WS and MS)	FINO (da Costa and others, 2016)	The members of the working groups of this organization are concerned about the quality of the work done by their team, work unit, others (Task orientation and climate of excellence)
		S4 (WS)	Lewis, 2003 Bachrach and others (2017)	Our team has a well agreed and well-focused line of work (Coordination)
	CWa	S1 (WS) S2 (WS)	CoPsoQ istas 21 (version 1.5) (Moncada et al., 2014)	Is your opinion, taken into account when you are assigned tasks?
		S4 (WS)	Ayestarán et al. (2006)	We have been able to adapt well to the different work schedules of the team members (Adaptation)
	EPs	S1 (WS) S2 (WS)	CoPsoQ istas 21 (version 1.5) (Moncada et al., 2014)	Is the distribution of tasks irregular, and causes work to accumulate? Does your job require you to hide your emotions?
	LpB	S1 (WS) S2 (WS)	CoPsoQ istas 21 (version 1.5) (Moncada et al., 2014)	At work, do you feel that you are part of a group? (Peer support) Do your immediate supervisors communicate well with workers? (Quality leadership)
		S3 (MS)	Castro Solano and others, 2004 (based on Bass) in Nader 2010	He is willing to instruct or teach us whenever we need it (Transformational Leadership); Whenever you think it is necessary, you can negotiate with me what you will get in return for your work (Transactional Leadership).
		S4 (WS)	Molero et al., 2007 (based on Bass) in Montano and others (2017)	To what extent does the team coordinator promote the participation of the other members in the decisions made by the team? The team coordinator emphasizes the importance of having a collective sense of the mission to be accomplished (Transformational Leadership)
			Ayestarán and others (2006)	The team coordinator emphasizes the importance of having a collective sense of the mission to be accomplished (Empowerment)
			Carson and others (2007)	How much does the team rely on the following people for leadership? (Shared Leadership)
Mesosocial	LeR	S1 (WS) S2 (WS)	Emotional labor (Zaft, et al., 1999 en Ortiz et al., 2012)	How often in your job do you have to display pleasant emotions towards clients (i. e. joy? (Positive emotions). How often in your job, do you have to display unpleasant emotions towards clients (i. e. strictness or anger if rules are not followed)? (Negative emotions). How often are you required to display neither positive nor negative emotions towards cli i. e. showing impartiality)? (Neutral emotions). How often is it necessary in your job to put yourself into your client's place (Empathy and sensibility)?. How often do you have to show emotions that do not correspond to your actual emotions? (Emotional Dissonance). You can decide yourself the amount of time you spend with a user,

			regardless of the user's needs? (Control of Interaction)
LR	S3 (WS and MS)	FINO (da Costa and others 2016)	The characteristics of the jobs in this organization, implies that people: Take challenges in performing their tasks (Complexity and role challenge); decide how to do their job (Role Autonomy)
CSO	S1 (WS) S2 (WS)	ODQ (Bass and Avolio, 1992 in Nader, 2006)	Stories are shared about the challenges that were overcome (Transformational Culture) You get what you earn, no more, no less (Transactional Culture) In this organization: collaboration between work groups is reinforced" (Integration)
	S3 (WS and MS)	FINO (da Costa and others, 2016)	The characteristics of the jobs in this organization imply that people has resources (physical space, documentary, others...) to carry out the tasks effectively are available (Resources)
LpO	S3 (WS and MS)	FINO (da Costa and others, 2016)	Regarding their employees, this organization's management (bosses, superiors, others) has a relationship of mutual trust (Positive Leadership). The people in charge (bosses, superiors, others) of this organization promote: action on the part of the workers, even though they may make a mistake (Innovation Leadership)
