# Supplementary material for: Belonging and Social Integration as Factors of Well-Being in Latin America and Latin Europe Organizations
Source: Front Psychol. 2020 Dec 9;11:604412. doi: 10.3389/fpsyg.2020.604412 (PMC7756150; doi:10.3389/fpsyg.2020.604412)
Supplement: Supplementary file 3 [file Table_3.pdf]

Belonging and Social Integration as Factors of Well-Being in Latin America and Latin Europe Organizations

Frontiers of Psychology

Silvia da Costa[1*], Edurne Martínez-Moreno[1], Virginia Díaz[1], Daniel Hermosilla[1], Alberto Amutio[2], Sonia Padoan[1], Doris Méndez[4], Gabriela Etchebehere[5], Alejandro Torres[6], Saioa Telletxea[3] and Silvia García Mazzieri[7]

[1]Department of Social Psychology, Faculty of Psychology, University of the Basque Country, San Sebastian, Spain

[2]Departament of Social Psychology, Faculty of Labour Relations and Social Work, University of the Basque Country, Leioa, Spain

[3]Departament of Social Psychology, Faculty of Labour Relations and Social Work, University of the Basque Country, Vitoria, Spain

[4]Departament of Psychology, Faculty of Psychology, University of Talca, Talca, Chile

[5]Institute of Psychology, Education and Human Development, Faculty of Psychology, University of the Oriental Republic of Uruguay, Montevideo, Uruguay

[6]Argentine National Defense University, Argentina

[7]Departament of Psychology, Regional Faculty of the National Technological University, Trenque Lauquen, Argentina

Corresponding author: Silvia da Costa e-mail: silviacristina.dacosta@ehu.eus

On line resources 3, Sociodemographic data of the participating sample by country in study 1

Socio-demographic variables study 1

Sample	Age	**Sex** Bevel point 1 = men, 2 = women	Civil status	Children	Besides working, do you study?	Study level	Current sector	Years in the sector	Job role<br>In the sector	Job role<br>At the time of the survey	**Time in your organization** (in the moment of the survey, 2014)	Kind of contract	**According to the methodology**	**Intention to stay** (in the organization long-term) **YES**
CHILE Santiago, Valparaíso, south-central region and Osorno<br><br>*N* =	19 to 66 years *M* = 34,08 *DT* = 11,85	*M* = 1,66, *DT* = .47 66,4 % women	58% single, 33% married, 2,3% separated, 2,0% divorced, 1,5%. 3% nr/dk	50% YES	96% YES	36,9% High school, 24,4% Graduate, 17,6% Postgraduate, 16,8% Secondary studies, < 4% Master’s and doctor’s degree	74% Educative, 21,3% Social intervention, 4,1% both sectors	M =, DT =	4.5% director / managerial position, 46.6% Pedagogue, 18% administrative, 13.5% Psychologist; 7.5% teacher, 6% others, 2.3% social educator,> 1% lawyer, service assistant	3.8% director / managerial position. 42.7% pedagogue, 18% administrative; 14.5% Psychologists, 9.9% professor; 4.6% social educator and others; > 1% cleaning assistant and educational assistant	10,5% < 10 years, >10%, 5 years, > 80% les than 5 years	35% permanent position, 13.4% temporary / provisional / substitute; 18.4% replacement / interim / substitute, 24% other	47% agree, 18.9% somewhat agree, 14.4 strongly agree, 10.7% fully agree, 4.5% not agree at all	64,3%
SPAIN (BC) Alaba/Gazteiz, Bilbao/Bilbo and Guipúzcoa/Gipuzkoa<br><br>*N* =	25 to 60 years *M* = 45,01 *DT* = 9,07	*M* = 1.79, *DT* = .415 78,5 % women	67,5% married, 23,6% single, 4,9% divorced, > 1% separated, 3,3% nr/dk	71% YES	73.5% NO	62% Graduate, 30.4% Bachelor's degree, 5.8% Master's degree, > 1% PhD	72% Educational, 26% Social intervention, 2.7% both sectors	*M* = 20,02, *DT* = 10,23	11.2% Director/Managerial position, 58.7% Teacher, 18.7% social educator, 2.4% Pedagogue, 2.4% Administrative ; 1.6% Administrative Assistant,	5.5% Director/Managerial position, 63.2% Teacher, 22.1% Social Educator, 1.8% Psychologist, Administrative and Social Worker respectively,	42.4% >10 years, 63.3% >5 years and 4.9% incorporation year survey.	70% permanent post; 14% temporary/provisional/substitute; 10.7% replacement/interim/substitute; 3.7% filling vacancy, 1.5% other	35.2% strongly agree, 33% agree, 18.6% total agree, 11.4% somewhat agree, 1.1% do not agree	79%

									1.2% Psychologist, > 1% Cleaning Assistant, Lawyer, Social Worker 2% others	1.1% Pedagogue. > 1% Cleaning Assistant, Lawyer and Tutor Teacher. 1.5% others				
Uruguay Montevideo Interior of the country *N* =	22 to 69 years *M* = 43.56 *DT* = 9.73	*M* = 1,91, *DT* = 91% women and 8,8% men	45% married, 21% single, 18% divorced, 3.6% widowed, 3.1% separated, 3.3% other (cohabitation, couple, free union) 6.1 nr/dk	76,8 % YES	60,5% NO	75,1% Graduate, 16,9% Bachelor's degree, 4,8% Master's degree	90.8% Educational, 1.9% Social intervention, 7.4% both sectors	*M* = 18,23, *DT* = 9,79	8.1 Director (12.6 management position), 67% Teacher, 8.4% Psychologist, 1.4% Pedagogue, 2.3% Social educator, 1.8% Social worker, 1.2% Administrative, 2.1% Assistant; 3% others	7.2 Director (10.1 management position), 74.3% Teacher, 7.4% Psychologist, 1.2% Pedagogue, 1.8% Social Worker, 1.4% Social Educator, less than 1% Administrative and equal Administrative Assistant; 2% others	>10 years with the current organization at the time of the survey (2015) (32.7%) > 5 years (48.4%), 12.6% had joined that year	78.6% permanent post, 15.3% temporary/provisional/substitute, 3.3% replacement/interim/substitute, less than 1% other	46% agree; somewhat agree and strongly agree 20% respectively; 9% total agree; 5% not agree	58%
