# Supplementary material for: Belonging and Social Integration as Factors of Well-Being in Latin America and Latin Europe Organizations
Source: Front Psychol. 2020 Dec 9;11:604412. doi: 10.3389/fpsyg.2020.604412 (PMC7756150; doi:10.3389/fpsyg.2020.604412)
Supplement: Supplementary file 4 [file Table_4.pdf]

Belonging and Social Integration as Factors of Well-Being in Latin America and Latin Europe Organizations

Frontiers of Psychology

Silvia da Costa[1*], Edurne Martínez-Moreno[1], Virginia Díaz[1], Daniel Hermosilla[1], Alberto Amutio[2], Sonia Padoan[1], Doris Méndez[4], Gabriela Etchebehere[5], Alejandro Torres[6], Saioa Telletxea[3] and Silvia García Mazzieri[7]

[1]Department of Social Psychology, Faculty of Psychology, University of the Basque Country, San Sebastian, Spain

[2]Departament of Social Psychology, Faculty of Labour Relations and Social Work, University of the Basque Country, Leioa, Spain

[3]Departament of Social Psychology, Faculty of Labour Relations and Social Work, University of the Basque Country, Vitoria, Spain

[4]Departament of Psychology, Faculty of Psychology, University of Talca, Talca, Chile

[5]Institute of Psychology, Education and Human Development, Faculty of Psychology, University of the Oriental Republic of Uruguay, Montevideo, Uruguay

[6]Argentine National Defense University, Argentina

[7]Departament of Psychology, Regional Faculty of the National Technological University, Trenque Lauquen, Argentina

Corresponding author: Silvia da Costa e-mail: silviacristina.dacosta@ehu.eus

On line resources 4, the first table shows the relationship between relationship between psychological well-being and other forms of well-being by nation, the second table shows the relationship between individual and microsocial level predictor variables with well-being by country, finally, the third table shows the same relationship at the mesosocial level by country participating in **study 1** of this research.

Relationship between psychological well-being and other forms of well-being by nation

Variables	Chile				Spain				Uruguay			
	n	Mean	*DT*	*r*	*n*	Mean	*DT*	*r*	n	Mean	*DT*	*r*
BSCs	338	43.88	8.88	34***	284	48.28	6.55	.44***	676	44.56	8.13	51***
QLLH	338	53.87	7.52	37***	279	55.09	7.95	.49***	651	52.35	10.36	.58***
AHW positive	360	25.97	7.19	39***	296	21.74	6.40	.45***	705	22.87	7.18	.49***
AHW negative	360	8.908	6.39	-.36***	295	6.786	5.45	-.24**	704	9.68	6.56	-.37 ***
SWL	351	38.99	6.61	.41***	284	39.17	5.44	.65 ***	699	38.03	7.05	.64***
EPWB	353	77.82	13.71	-	296	79.53	11.54	-	703	83.84	14.12	-

Note: BSCs = Behavioral, somatic and cognitive reactions to stress; QLLH = Quality of Life linked to health; AHWB = Affective hedonic view of subjective Well-Being; SWL = Cognitive hedonic view of subjective Well-Being or satisfactorial with life; EPWB = Eudaimonic vision or psychological Well-Being and personal optimal development. *** $p<.001$

Relationship between individual and microsocial level predictor variables with well-being by country

Variables	Chile (*n* = 333)							Spain (*n* = 284)							Uruguay (*n* = 688)						
	1	2	3	4	5	6	7	1	2	3	4	5	6	7	1	2	3	4	5	6	7
Gender	-.09*	-.13**	-.07	.01	-.05	-.008	.03	-.07	.03	-.06	-.11*	.02	-.01	-.09	-.01	-.06	.02	-.01	.02	-.07*	.02
EC	-.06	-.04	.21**	.15**	.05	-.009	.07	-.14**	-.19**	.16**	.20**	-.009	.016	.13*	-.12**	-.02	.21**	.14**	.05	.17**	.18**
OVC	.09	.07	.23**	.04	.13**	.010	.18**	.04	-.21**	-.04	.04	-.05	.04	.14**	.11**	.15**	.26**	-.05	.20**	.19**	.30**
TVU	.19**	.15**	.19**	-.15**	.21**	.10*	.27**	.14**	-.20**	.005	-.002	.004	.03	.23**	.05	.16**	.26**	-.06	.20**	.15**	.25**
SO	.05	.07	.04	.04	.001	-.19**	-.02	.08	-.02	-.02	-.06	.03	-.03	.04	.008	-.04	-.10**	-.04	-.05	-.03	-.04
AM	.25**	.24**	.23**	-.16**	.25**	.21**	.35**	.19**	.20**	.37**	-.14*	.34**	.31**	.25**	.24**	.29**	.32**	-.25**	.36**	.30**	.28**
IS	.22**	.18**	.22**	-.12*	.23**	.23**	.20**	.05	-.002	-.02	-.11*	.05	.04	-.007	.10**	.14**	.23**	-.21**	.28**	.18**	.15**
EPs	-.30**	-.33**	-.29**	.36**	-.41**	-.30**	-.17**	-.48**	-.38**	-.29**	.48**	-.48**	-.37**	-.29**	-.40**	-.44**	-.33**	.38**	-.45**	-.26**	-.30**
CWa	.06	.24**	.25**	-.19**	.28**	.18**	.29**	.10*	.11*	.38**	-.14**	.34**	.36**	.32**	.27**	.40**	.50**	-.33**	.53**	.51**	.35**
LpB	.07	.24**	.26**	-.21**	.30**	.17**	.22**	.35**	.37**	.48**	-.44**	.58**	.42**	.36**	.23**	.31**	.42**	-.34**	.49**	.29**	.32**

Note. 1 = BSCs; 2 = CVRS; 3 = AHWB positive; 4 = AHWB negative; 5 = Scale of affection; 6 = SWL; 7 = EPWB. Gender = 1 = women, 2 = men; CE = Emotional Creativity; OVC = Openness to change values; TVU = Values of transcendence Universalism; SO = Seniority in the organization; AM = Agreement with the methodology in the workplace; IS = Intention to stay; Eps = Excess of psychological demands at work or work stress; CWa = Control over work, role autonomy; LpB = Leadership that reinforces participation and belonging. ** $p<.01$ *$p<.05$

Relationship between mesosocial level predictor variables with well-being by country

Variables	Chile (*n* = 350)							Spain (*n* = 283)							Uruguay (*n* = 685)						
	1	2	3	4	5	6	7	1	2	3	4	5	6	7	1	2	3	4	5	6	7
LeR	-.17**	-.11*	-.12*	-.02	.18**	-.08	-.20**	-.20	.06	-.11*	.008	.22**	-.03	-.17**	-.29**	-.13**	-.26**	-.12**	.29**	-.12**	-.28**
LeR NE	-.16**	-.21**	-.19**	-.20*	.22**	-.10*	-.19**	-.11*	-.11*	-.24**	-.16**	.22**	-.17**	-.16**	-.32**	-.26**	-.33**	-.24**	.30**	-.21**	-.35**
LeR NEE	-.10*	-.07	-.09*	-.04	.11*	.003	-.13**	-.06	.02	-.12*	-.05	.16**	-.06	-.17**	-.21**	-.11**	-.19**	-.07*	.23**	-.08*	-.22**
LeR PE	-.10*	-.04	-.04	-.003	.07	-.10*	-.10*	-.04	.16**	.04	.13*	.10	.09	-.05	-.07*	.05	-.004	.06	.06	.01	-.09*
LeR ED	-.22**	-.11*	-.21**	-.11*	.23**	-.15**	-.21**	-.14*	-.08	-.21**	-.13*	.22**	-.18**	-.25**	-.30**	-.23**	-.34**	-.22**	.33**	-.18**	-.29**
LeR SandE	.01	.12*	.16**	.19**	-.07	.05	-.01	-.009	.15**	.14**	.19**	-.01	.24**	.09	-.007	.08*	.02	.08*	.03	.04	.006
LeR CINT	.11*	.12**	.20**	.20**	-.10*	.11*	.07	.17**	.27**	.32**	.32**	-.17**	.28**	.18**	.15**	.20**	.20**	.18**	-.16**	.19**	.13**
CSO Transf.	.05	.10*	.25**	.24**	-.15**	.03	.19**	.08	.28**	.37**	.37**	-.21**	.22**	-.001	.17**	.26**	.36**	.35**	-.21**	.22**	.22**
CSO Transac	-.12*	-.05	-.02	.05	.09*	-.10*	-.04	-.21**	-.20**	-.35**	-.34**	.22**	-.20**	-.15**	-.21**	-.10**	-.25**	-.13*	.28**	-.15**	-.23**

Nota: 1 = BSCs; 2 = CVRS; 3 = AHWB positive; 4 = AHWB negative; 5 = Scale of affection; 6 = SWL; 7 = EPWB. LeR = emotional labor role; LeR NE = negative expression of emotions in the labor role; LeR NEE = neutral expression of emotions in the labor role ; LeR PE = positive expression of emotions in the labor role ; LeR ED = emotional dissonance in the labor role ; LeR SE = sensitivity and empathy in the labor role ; LeR CINT = control of interaction in the labor role ; CSO = Culture and organizational structure that reinforces participation and integration (transformational and transactional). ** p<.01 *p<.05
