# Supplementary material for: Belonging and Social Integration as Factors of Well-Being in Latin America and Latin Europe Organizations
Source: Front Psychol. 2020 Dec 9;11:604412. doi: 10.3389/fpsyg.2020.604412 (PMC7756150; doi:10.3389/fpsyg.2020.604412)
Supplement: Supplementary file 5 [file Table_5.pdf]

Belonging and Social Integration as Factors of Well-Being in Latin America and Latin Europe Organizations

Frontiers of Psychology

Silvia da Costa[1*], Edurne Martínez-Moreno[1], Virginia Díaz[1], Daniel Hermosilla[1], Alberto Amutio[2], Sonia Padoan[1], Doris Méndez[4], Gabriela Etchebehere[5], Alejandro Torres[6], Saioa Telletxea[3] and Silvia García Mazzieri[7]

[1]Department of Social Psychology, Faculty of Psychology, University of the Basque Country, San Sebastian, Spain

[2]Departament of Social Psychology, Faculty of Labour Relations and Social Work, University of the Basque Country, Leioa, Spain

[3]Departament of Social Psychology, Faculty of Labour Relations and Social Work, University of the Basque Country, Vitoria, Spain

[4]Departament of Psychology, Faculty of Psychology, University of Talca, Talca, Chile

[5]Institute of Psychology, Education and Human Development, Faculty of Psychology, University of the Oriental Republic of Uruguay, Montevideo, Uruguay

[6]Argentine National Defense University, Argentina

[7]Departament of Psychology, Regional Faculty of the National Technological University, Trenque Lauquen, Argentina

Corresponding author: Silvia da Costa e-mail: silviacristina.dacosta@ehu.eus

On line resources 5, Sociodemographic data of the participating sample by country in **study 2**. The variables used specifically in this study are marked in gray.

Socio-demographic variables Group 1 (Talca)

Sample	Age	**Sex** Biserial point 1 = men, 2 = women	Civil State	Children (YES)	Besides working, do you study? (YES)	Level of study	Current sector Education Social Intervention	Years in the sector	Job role (At the time of the survey)	Current medical leave or sick leave	**Time in your organization (at the time of the survey, 2014)**	Type of contract	**Agreement with the methodology**	**Intention of permanence (in the organization in the long term)** YES
CHILE Talca School	23 to 61 years *M* = 39,14 *DT* = 10,23	*M* = 1,75, *DT* = .439 75% women	51.4% single, 40.5% married, 2.7% divorced; 5.4% widowed	60%	82%	83% Graduate, 8.7% Master/Master's Degree	Educational	*M* = 11,30, *DT* = 10,16	13.5% management position, 73% professor, 11.7% educational assistant, 2.7% administrative	67% due to physical illness, 33.3% due to occupational stress	30% < 10 years	59.5% permanent position, 37.8% indefinite, 2.7% substitute	59.5% agree, 24.3% somewhat agree, 8.1% total agree, 5.4% do not agree, 2.7% very agree	57,8%
