# Supplementary material for: Belonging and Social Integration as Factors of Well-Being in Latin America and Latin Europe Organizations
Source: Front Psychol. 2020 Dec 9;11:604412. doi: 10.3389/fpsyg.2020.604412 (PMC7756150; doi:10.3389/fpsyg.2020.604412)
Supplement: Supplementary file 6 [file Table_6.pdf]

Belonging and Social Integration as Factors of Well-Being in Latin America and Latin Europe Organizations

Frontiers of Psychology

Silvia da Costa[1*], Edurne Martínez-Moreno[1], Virginia Díaz[1], Daniel Hermosilla[1], Alberto Amutio[2], Sonia Padoan[1], Doris Méndez[4], Gabriela Etchebehere[5], Alejandro Torres[6], Saioa Telletxea[3] and Silvia García Mazzieri[7]

[1]Department of Social Psychology, Faculty of Psychology, University of the Basque Country, San Sebastian, Spain

[2]Departament of Social Psychology, Faculty of Labour Relations and Social Work, University of the Basque Country, Leioa, Spain

[3]Departament of Social Psychology, Faculty of Labour Relations and Social Work, University of the Basque Country, Vitoria, Spain

[4]Departament of Psychology, Faculty of Psychology, University of Talca, Talca, Chile

[5]Institute of Psychology, Education and Human Development, Faculty of Psychology, University of the Oriental Republic of Uruguay, Montevideo, Uruguay

[6]Argentine National Defense University, Argentina

[7]Departament of Psychology, Regional Faculty of the National Technological University, Trenque Lauquen, Argentina

Corresponding author: Silvia da Costa e-mail: silviacristina.dacosta@ehu.eus

On line resources 6, general and specific socio-demographic data by country participating in the **study 3.** The variables used specifically in this study are marked in gray and in **bold relief.**

General socio-demographic

Sample	Sex	Age	Educational Level	Relevant data on members and the organisation
Workers<br><br>*N* = 683	50,9% men; 49,1 women *M* = 1.49, *DT* = .500	*M* = 36,27, *DT* = 10,70	79% tertiary	> 500 employees (60.1%); between 10 and 50 (20.5%) Civil service (56.5%), private (41.2%), third sector (2.4%). Service sector (78.1%), Industrial (15.2%), Agricultural (5.7%), <1% transport, commercial aviation, other. **Seniority >10 years in the current organization (27.8%), > 6 months and up to 2 years (21.5%). Permanent contract (74%).** Worked in two or more departments (56%) and held two or more positions during working life (55%). Worked continuously (66.7%), from Monday to Saturday (15.7%). From Monday to Friday (57.8%). Fixed salary (78.1%), a fixed part and a variable part (17.1%). Has not been promoted in category/position or professional group since joining the organisation (51.6%). The work that he does corresponds to the professional category/position in the ladder (58.1%); his work is above what is recognized in the salary (30.8%), it is below (4%), he does not know (5.5%). Their work is well paid (71.4%). He does not hold a management position (76.6%), yes (23.4%). Has been on sick leave in the last year (56.8%)
Military<br><br>*N* = 315	85,7% men; 12,1% women *M* = 1.12, *DT* = 3.29	*M* = 23.52, *DT* = 2.39	3rd and 4th year students (50 and 50) of a Military Academy, in positions of leadership in relation to other students	Arsenals 13% (*n* = 36); Quartermasters 7% (*n* =21); Infantry 31% (*n* = 89) ; Artillery 13% (*n* = 13) ; Communications 12% (*n* = 35) ; Engineering 12% (*n* = 33) ; Cavalry 11% (*n* = 31)

Socio-demographic variables by country

Sample	Sex	Age	Educational Level	Relevant data on members and the organisation
Argentina<br><br>*N* = 50	*M* = 1,34, *DT* = 1,479 66% men	22 to 53 years, *M* = 29,92, *DT* = 7,395	66% up to tertiary education, 34% tertiary education	> 11 to 50 employees (46%), up to 10 (36%), 101 to 500 (18%). Private function (90%), public (8%), third sector (2%). Agricultural sector (92%), service (8%). **Seniority > 2 and up to 5 years (34%), between one month and up to 6 months (22%), > 6 months and up to 2 years (18%), > 5 and up to 10 years, more than 10 years (10%), less than 30 days (6%). Fixed contract (80%).** Worked in two or more departments and occupied two or more positions during your working life (90% NOT respectively). Working day from Monday to Saturday (80%), from Monday to Friday (14%). Fixed salary (62%), a fixed part and a variable part (34%). Not promoted in category/position or professional group since joining the organization (78%). The work you do corresponds to the professional category/position in the ladder (64%); your work is above what is recognised in your salary (12%), it is below (4%), you do not know it (6%). Their work is well paid (66%). He does not hold a management position (88%), yes (12%). You have been on sick leave in the last year (74% NO)
Brazil<br><br>*N* = 316	*M* = 1.69, *SD* = .490 60% women	18 a 75 years, *M* = 35,36, *SD* = 9,996	81% tertiary education	<500 employees (43.2), 101 to 500 (28%), 11 to 50 (13.6), 51 to 100 and up to 10 <8%. Private function (71%), public (25%), third sector (5%). Service sector (66%), industry (31%), agriculture (3%). **Seniority >6 months and up to 2 years (27%), >2 years and up to 5 years (25%), >5 years and up to 10 years (20%), >10 years (18%), <1 month and up to 6 months (9%), <30 days (2%). Permanent contract (76%).** Worked in two or more departments and held two or more positions during their working life (73% YES). Continuous working day from Monday to Friday (66%). Fixed salary (81%). Was promoted in category/position or professional group since joining the organization (89%). The work you do corresponds to the professional category/position in the ladder (52%), is above what is recognised in your salary (38.5%), is below (2.9%), does not know (6.6%). His work is well paid (83%). He does not hold a management position (62%), not (38%). Has been on sick leave during the last year (71% YES)
Experts: Chile and Spain<br><br>*N* = 15	M = 1,33, SD = .488 67% men	23 to 66 years, *M* = 42,20, *SD* = 11,55	100% tertiary education	Up to 10 employees (44.4%), >500 (33.3%). Private function (92%). Service sector (67%), agriculture (n = 2), commercial aviation (n = 1). **Seniority > 2 and up to 5 years (49%), >10 years (30%), > 5 and up to 10 years (20%), >6 months and up to 2 years (10%). Fixed contract (80%).** Worked in two or more departments (56% YES) and occupied two or more positions during their working life (62% NO). Continuous working day from Monday to Friday (45.5%); exceptionally Saturday, Sunday and public holidays (36.4%), from Monday to Saturday (18%). Fixed salary

				(66.7%); fixed part/variable part (33.3%). Was promoted from category/position or professional group since joining the organisation (56% NO). Was promoted in category/position or professional group since joining the organization (56% NO). The work you do corresponds to the professional category/position in the ladder (89%), is above what is recognised in your salary (n = 1). Your work is well paid (78%). You do not hold a management position (78%), no (22%). You have been on sick leave in the last year (67% NO)
Spain<br><br>*N* = 269	*M* = 1,46, *SD* = .500 54% men	24 to 67 years<br>*M* = 43,35, *SD* = 11,05	99% tertiary education	>500 employees (55.5%), from 51 to 100 (20%), from 101 to 500 (12%) and up to 10 (4.5%) Public service (63.4%), private (36.6%). Service sector (94%), industry (6%). **Seniority >10 years (51%), >2 years and up to 5 years (19%), >5 years and up to 10 years (13%), >6 months and up to 2 years (10%), <30 days (7%). Permanent contract (62%), temporary (33%).** Worked in two or more departments and occupied two or more positions during their working life (64% and 66% NOT respectively). Continuous working day from Monday to Friday (60%), exceptionally Saturdays, Sundays and public holidays (24%). Fixed salary (85%). Has been promoted in category/position or professional group since joining the organization (61%). The work you do corresponds to the professional category/position in the ladder (66%), is above what is recognised in your salary (23%), is below (7.5%), does not know (3.8%). His work is well paid (53% NO). He does not hold any management position (79% NO). You have been on sick leave during the last year (56% NO)
Mexico<br><br>*N* = 135	*M* = 1.41, *SD* = .493 59% men	18 to 57 years, *M* = 32,40, *SD* = 8,28	62% tertiary education	> 11 to 50 employees (41.5%), >500 employees (24.4%), 101 to 500 (23%), up to 10 (6.7%), 51 to 100 (4.4%) Private function (70%), public (24.4%), third sector (5.2%). Service sector (48.9%), industry (45.2%), >1% aviation transport and others. **Seniority > 6 months and up to 2 years (32.6%), > 2 and up to 5 years (32%), between 1 month and up to 6 months (15.6%), > 5 and up to 10 years (10.4%), more than 10 years (9.6%). Permanent contract (83%).** Worked in two or more departments and occupied two or more positions during their working life (50% NOT respectively). Working day from Monday to Saturday (50%), from Monday to Friday (50%). Fixed salary (50%), a fixed part and a variable part (50%). You have not been promoted in any category/position or professional group since you joined the organization (50% NO). The work you do corresponds to the professional category/position in the ladder (100% YES). Your work is well paid (50%). You do not hold a management position (88%), yes (12%). You have been on sick leave during the last year (100% NO)
