# Supplementary material for: Belonging and Social Integration as Factors of Well-Being in Latin America and Latin Europe Organizations
Source: Front Psychol. 2020 Dec 9;11:604412. doi: 10.3389/fpsyg.2020.604412 (PMC7756150; doi:10.3389/fpsyg.2020.604412)
Supplement: Supplementary file 7 [file Table_7.pdf]

Belonging and Social Integration as Factors of Well-Being in Latin America and Latin Europe Organizations

Frontiers of Psychology

Silvia da Costa[1*], Edurne Martínez-Moreno[1], Virginia Díaz[1], Daniel Hermosilla[1], Alberto Amutio[2], Sonia Padoan[1], Doris Méndez[4], Gabriela Etchebehere[5], Alejandro Torres[6], Saioa Telletxea[3] and Silvia García Mazzieri[7]

[1]Department of Social Psychology, Faculty of Psychology, University of the Basque Country, San Sebastian, Spain

[2]Departament of Social Psychology, Faculty of Labour Relations and Social Work, University of the Basque Country, Leioa, Spain

[3]Departament of Social Psychology, Faculty of Labour Relations and Social Work, University of the Basque Country, Vitoria, Spain

[4]Departament of Psychology, Faculty of Psychology, University of Talca, Talca, Chile

[5]Institute of Psychology, Education and Human Development, Faculty of Psychology, University of the Oriental Republic of Uruguay, Montevideo, Uruguay

[6]Argentine National Defense University, Argentina

[7]Departament of Psychology, Regional Faculty of the National Technological University, Trenque Lauquen, Argentina

Corresponding author: Silvia da Costa e-mail: silviacristina.dacosta@ehu.eus

On line resources 7, Sociodemographic data of the participating sample by country in **study 4.** The variables used specifically in this study are marked in gray.

Socio-demographic variables

-	Age (years)		**Sex**<br>Biserial poinr<br>1 = men, 2 = women	Level of study	Current sector	Job role<br><br>At the time of the survey	**Time in the organization**
Spain	22 to 59 years		63.4% (*n* = 52) women<br>36.6% (*n* = 30) men	University studies 67% (n = 55)<br>Senior technician 12% (n = 10)<br>Average technician 11% (n = 9)<br>PhD 8.5% (n = 7)<br>Primary education 1.2% (n = 1)	Service sector 51.2% (k = 5)<br>Production sector 48.8% (k = 3)	Basic workers 53.8% (n = 43)<br>Middle management 35.0% (n = 28)<br>Management team 11.3% (n = 9)	< 5 years 37.8% (*n* = 31)<br>6-15 years 42.7% (*n* = 35)<br>16-25 years 11.0% (*n* = 9)<br>>26 years 8.5 % (*n* = 7)
	M = 35.88	*SD* = 9.15					
